# CSE1L promotes nuclear accumulation of transcriptional coactivator TAZ and enhances invasiveness of human cancer cells

**DOI:** 10.1016/j.jbc.2021.100803

**Published:** 2021-05-20

**Authors:** Shunta Nagashima, Junichi Maruyama, Kaori Honda, Yasumitsu Kondoh, Hiroyuki Osada, Makiko Nawa, Ken-ichi Nakahama, Mari Ishigami-Yuasa, Hiroyuki Kagechika, Haruhiko Sugimura, Hiroaki Iwasa, Kyoko Arimoto-Matsuzaki, Hiroshi Nishina, Yutaka Hata

**Affiliations:** 1 Department of Medical Biochemistry, Graduate School of Medical and Dental Sciences, Tokyo Medical and Dental University, Tokyo, Japan; 2 Chemical Biology Research Group, RIKEN Center for Sustainable Resource Science, Saitama, Japan; 3 Laboratory of Cytometry and Proteome Research in Nanken-Kyoten and RCC, Tokyo Medical and Dental University, Tokyo, Japan; 4 Department of Cellular Physiological Chemistry, Tokyo Medical and Dental University, Tokyo, Japan; 5 Chemical Biology Screening Center, Tokyo Medical and Dental University, Tokyo, Japan; 6 Institute of Biomaterials and Bioengineering, Tokyo Medical and Dental University, Tokyo, Japan; 7 Department of Tumor Pathology, Hamamatsu University School of Medicine, Hamamatsu, Japan; 8 Department of Developmental and Regenerative Biology, Medical Research Institute, Tokyo Medical and Dental University, Tokyo, Japan; 9 Center for Brain Integration Research, Tokyo Medical and Dental University, Tokyo, Japan

**Keywords:** CSE1L, Hippo pathway, nuclear transport, TAZ/WWTR1, APMSF, (p-amidinophenyl)methanesulfonyl fluoride hydrochloride, CAMTA1, calmodulin-binding transcription activator 1, CAS, cellular apoptosis susceptibility, CSE1L, chromosomal segregation 1 like, EHE, epithelioid hemangioendothelioma, FBS, fetal bovine serum, FRAP, fluorescence recovery after photobleaching, GFP-TAZ, GFP-tagged TAZ, HGNC, HUGO Gene Nomenclature Committee, LATS, large tumor suppressor, MTT, 3-[4,5-dimethylthiazol-2-yl]-2,5-diphenyl tetrazolium bromide, NLS, nuclear localization signal, TAZ, transcriptional coactivator with PDZ-binding motif, TI-4, TAZ inhibitor 4

## Abstract

The transcriptional coactivator with PDZ-binding motif (TAZ) (*WWTR1*) induces epithelial–mesenchymal transition and enhances drug resistance in multiple cancers. TAZ has been shown to interact with transcription factors in the nucleus, but when phosphorylated, translocates to the cytoplasm and is degraded through proteasomes. Here, we identified a compound TAZ inhibitor 4 (TI-4) that shifted TAZ localization to the cytoplasm independently of its phosphorylation. We used affinity beads to ascertain a putative target of TI-4, chromosomal segregation 1 like (CSE1L), which is known to be involved in the recycling of importin α and as a biomarker of cancer malignancy. We found that TI-4 suppressed TAZ-mediated transcription in a CSE1L-dependent manner. CSE1L overexpression increased nuclear levels of TAZ, whereas *CSE1L* silencing delayed its nuclear import. We also found *via* the *in vitro* coimmunoprecipitation experiments that TI-4 strengthened the interaction between CSE1L and importin α5 and blocked the binding of importin α5 to TAZ. *WWTR1* silencing attenuated CSE1L-promoted colony formation, motility, and invasiveness of human lung cancer and glioblastoma cells. Conversely, *CSE1L* silencing blocked TAZ-promoted colony formation, motility, and invasiveness in human lung cancer and glioblastoma cells. In human cancer tissues, the expression level of CSE1L was found to correlate with nuclear levels of TAZ. These findings support that CSE1L promotes the nuclear accumulation of TAZ and enhances malignancy in cancer cells.

The transcriptional coactivator with PDZ-binding motif (TAZ) (TAZ is widely used but as its official gene symbol is *WWTR1* (HUGO Gene Nomenclature Committee [HGNC] ID: HGNC 24042; Entrez Gene 25937), we used TAZ and *WWTR1* in this article for the protein [NP001161750.1] and the gene, respectively) was first identified as a protein that binds to 14-3-3 (1). The phosphorylation at serine 89 by large tumor suppressor (LATS) kinases triggers the interaction with 14-3-3, resulting in the cytoplasmic segregation of TAZ (2). Phosphorylation by LATS kinases also causes protein degradation (3, 4). Thus, TAZ is negatively regulated by LATS kinases, core kinases of the tumor suppressor Hippo pathway. In human cancers, the dysregulation of the Hippo pathway and *WWTR1* gene amplification lead to TAZ hyperactivation (5, 6). TAZ activation induces epithelial–mesenchymal transition, enhances drug resistance, confers stemness to cancer cells, and is associated with poor prognosis in cancers. Hence, TAZ is regarded as a potential target for cancer therapy.

We screened for TAZ inhibitors by means of a cell-based assay (7). We expressed GFP-tagged TAZ (GFP-TAZ) in human osteosarcoma U2OS cells, in which the Hippo pathway is activated and inactivated depending on the cell density. In U2OS cells plated at low density, GFP-TAZ is accumulated in the nucleus. We applied 18,606 small chemical compounds to the cells and obtained as putative TAZ inhibitors compounds that shifted GFP-TAZ to the cytoplasm. We further selected compounds (IBS000540, IBS001594, IBS015181, and IBS015625) that significantly suppressed TAZ/TEAD-driven luciferase reporter activity in HEK293FT cells. IBS000540, IBS001594, and IBS015625 increased phosphorylated TAZ and decreased unphosphorylated TAZ. Hence, these three compounds inhibited TAZ in the phosphorylation-dependent manner through the canonical Hippo pathway. We previously reported these three compounds as TAZ inhibitors (7). In contrast, IBS015181 did not increase the amount of phosphorylated TAZ. It implies that the effect of IBS015181 does not depend on the canonical Hippo pathway. In this article, we named IBS015181 (IUPAC name: 2,2-dichloro-*N*-(4-nitrophenyl)-3-phenylcyclopropane-1-carboxamide) as TAZ inhibitor 4 (TI-4) and characterized it.

Chromosomal segregation 1 like (CSE1L) (also called cellular apoptosis susceptibility (CAS) and exportin-2; according to its official gene symbol (HGNC 2431; Entrez Gene 1434), we used CSE1L to describe both the protein (NP001307) and the gene in this article) was identified as the gene that rendered human breast cancer MCF-7 cells resistant to immunotoxins and turned out to be homologous to yeast *CSE1* gene, which is involved in the regulation of chromatins (8, 9, 10). CSE1L binds to importin α in the presence of RanGTP and is required for the recycling of importin α (11, 12).

CSE1L is implicated in cell proliferation and apoptosis in human cancers (13). High expression of CSE1L is associated with a poor prognosis in cancers and is considered to be a prognostic marker (14, 15, 16, 17). However, the mechanism underlying of the oncogenic action of CSE1L is not fully understood.

We identified CSE1L as the target of TI-4. We revealed that CSE1L is involved in the regulation of the nuclear import of TAZ. Moreover, we demonstrated that CSE1L increases the nuclear TAZ and enhances malignancy in cancer cells.

## Results

### TI-4 shifts GFP-TAZ from the nucleus to the cytoplasm in U2OS cells but does not increase phosphorylated TAZ

**Figure 1:**
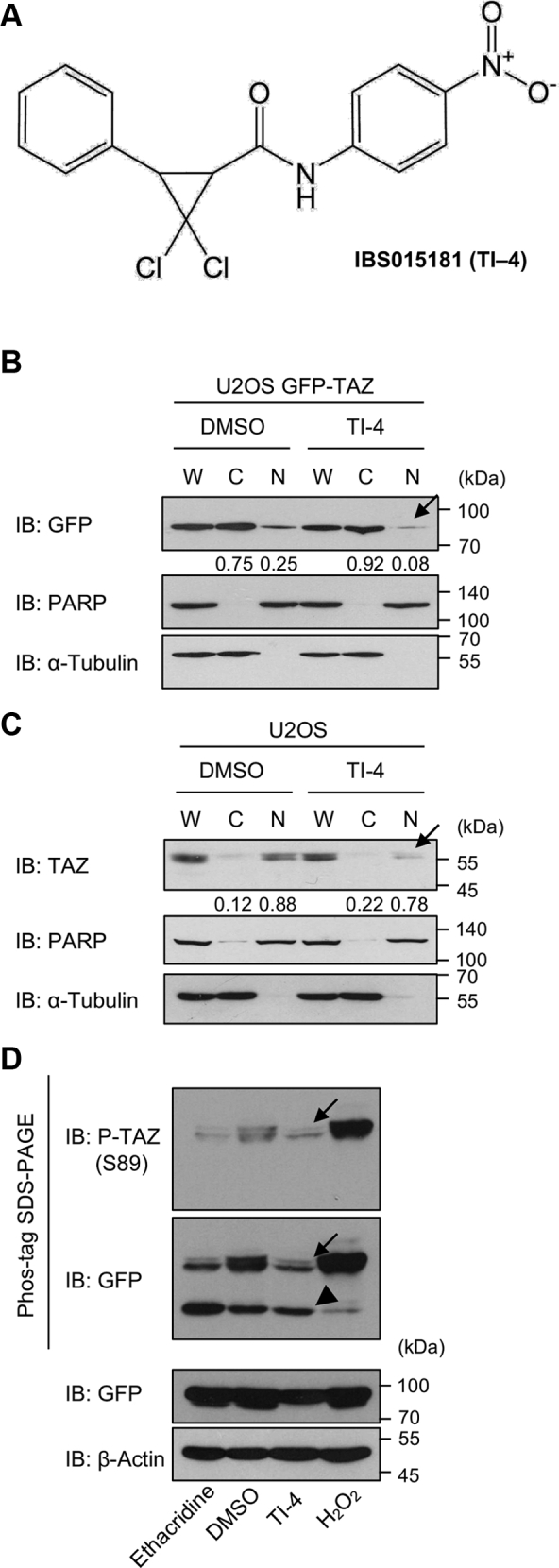
**The effect of TI-4 on the subcellular localization and the phosphorylation of TAZ.***A*, the structural formula of TI-4. *B*, U2OS cells expressing GFP-TAZ were plated at 5 × 10^5^ cells/10-cm dish and 24 h later, treated with DMSO or 10 μM TI-4. Forty-eight hours later, the subcellular fractionation was performed. The comparable amounts of the whole-cell lysate (W), the cytoplasmic fraction (C), and the nuclear fraction (N) were immunoblotted with the indicated antibodies. PARP and α-tubulin were used as the nuclear and cytoplasmic markers, respectively. TI-4 decreased the nuclear GFP-TAZ (an *arrow*). *C*, the subcellular fractionation was performed by using U2OS cells. Endogenous TAZ was detected and analyzed as in panel *B*. *D*, U2OS cells expressing GFP-TAZ were plated at 1 × 10^5^ cells/well in a 6-well plate and 24 h later, treated with DMSO or 10 μM TI-4. Twenty-four hours later, the whole-cell lysates were run on Phos-tag gels and immunoblotted with anti-phospho-Ser89 (P-TAZ(S89)) and GFP antibodies. As control, the cells were exposed to 5 μM ethacridine for 24 h or 1 mM H_2_O_2_ for 30 min. Ethacridine decreased phosphorylated TAZ and increased unphosphorylated TAZ (the first lane). H_2_O_2_ treatment increased phosphorylated TAZ and decreased the unphosphorylated TAZ (the fourth lane). The experiments in panels B, C, and D were repeated more than three times. DMSO, dimethyl sulfoxide; GFP-TAZ, GFP-tagged TAZ; PARP, poly (adp-ribose) polymerase; TAZ, transcriptional coactivator with PDZ-binding motif; TI-4, TAZ inhibitor 4.

The structure of TI-4 is not related to those of the previously reported TAZ inhibitors, IBS00540, IBS001594, and IBS015625 (Fig. 1*A*) (7). First, we confirmed the effect of TI-4 on the subcellular distribution of TAZ in U2OS cells expressing GFP-TAZ. TI-4 decreased the nuclear GFP-TAZ (Fig. 1*B*, an arrow). TI-4 also reduced endogenous TAZ in the nucleus in U2OS cells (Fig. 1*C*, an arrow). In the Phos-tag gel, TI-4 rather decreased the phosphorylated TAZ (Fig. 1*D*, arrows), which was detected with anti-phospho-TAZ (S89) antibody but did not increase the unphosphorylated TAZ (Fig. 1*D*, an arrowhead). We also applied TI-4 to the cells expressing GFP-TAZ S89A mutant (Fig. S1*A*). TI-4 shifted GFP-TAZ S89A to the cytoplasm, which further supports that the effect of TI-4 is independent of TAZ phosphorylation.

### TI-4 suppresses TEAD target genes in U2OS cells

**Figure 2:**
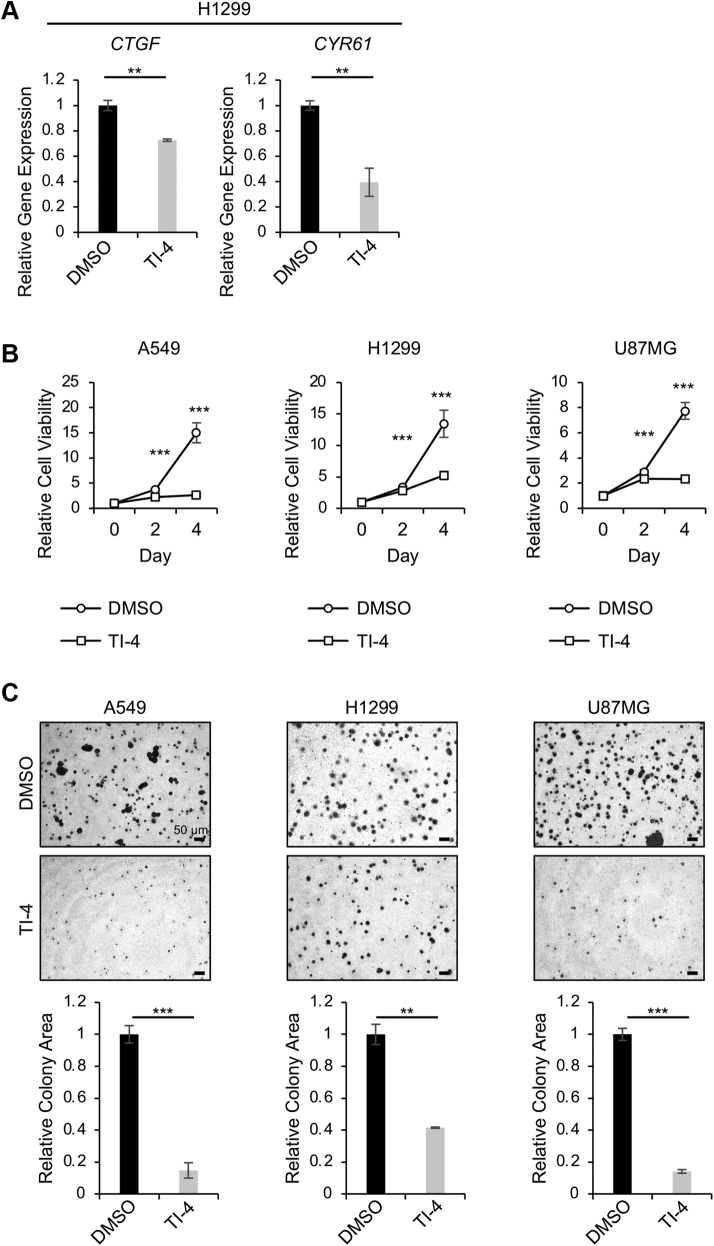
**The effect of TI-4 on cancer cells.***A*, H1299 cells were plated at 1 × 10^5^ cells/well in a 6-well plate. Twenty-four hours later, the cells were treated with DMSO or 10 μM TI-4 for 48 h. Total RNA was extracted, and quantitative RT-PCR was performed. The experiments were performed three times. *B*, A549, H1299, and U87MG cells were plated at 3000 cells/well in a 96-well plate. Twenty-four hours later, the cells were treated with DMSO or 10 μM TI-4 and cultured for 48 h and 96 h. The colorimetric assay was performed. The experiments were performed three times. *C*, A549, H1299, and U87MG cells were treated with DMSO or 10 μM TI-4. The cells were plated in the soft agar. On day 14, the colony area was evaluated with ImageJ. The mean value for DMSO-treated cells was set at 1.0. The experiments were performed three times. In panels *A*–*C*, data are shown as the mean ± SD. ∗∗*p* < 0.01; and ∗∗∗*p* < 0.001. The experiments were repeated three times. DMSO, dimethyl sulfoxide; TI-4, TAZ inhibitor 4.

In the previous study, we showed that TI-4 suppressed TAZ/TEAD-driven luciferase reporter activity in HEK293FT cells (7). In the reporter assay, we used the artificial reporter gene driven by eight repeats of the TEAD-responsive sequence. In this study, we examined the effect of TI-4 on endogenous TEAD target genes. quantitative RT-PCR demonstrated that TI-4 suppressed the expression of *CTGF* and *CYR61* in H1299 cells (Fig. 2*A*).

### TI-4 suppresses cell proliferation and soft agar colony formation of A549, H1299, and U87MG cells

As TAZ and TEAD play a role in the cancer development, we next examined the effect of TI-4 on cancer cells. We applied TI-4 to A549, H1299, and U87MG cells. We previously confirmed that *WWTR1* silencing compromises the cell viability in these cancer cells (7). Therefore, if TI-4 is a TAZ inhibitor, it should suppress the viability of these cancer cells. Indeed, 3-[4,5-dimethylthiazol-2-yl]-2,5-diphenyl tetrazolium bromide (MTT) assays showed that TI-4 suppressed cell proliferation in these cells (Fig. 2*B*). TI-4 also blocked colony formation in the soft agar (Fig. 2*C*). These findings support that TI-4 inhibits TAZ/TEAD target genes and exhibits the anticancer effect.

### CSE1L binds to TI-4

**Figure 3:**
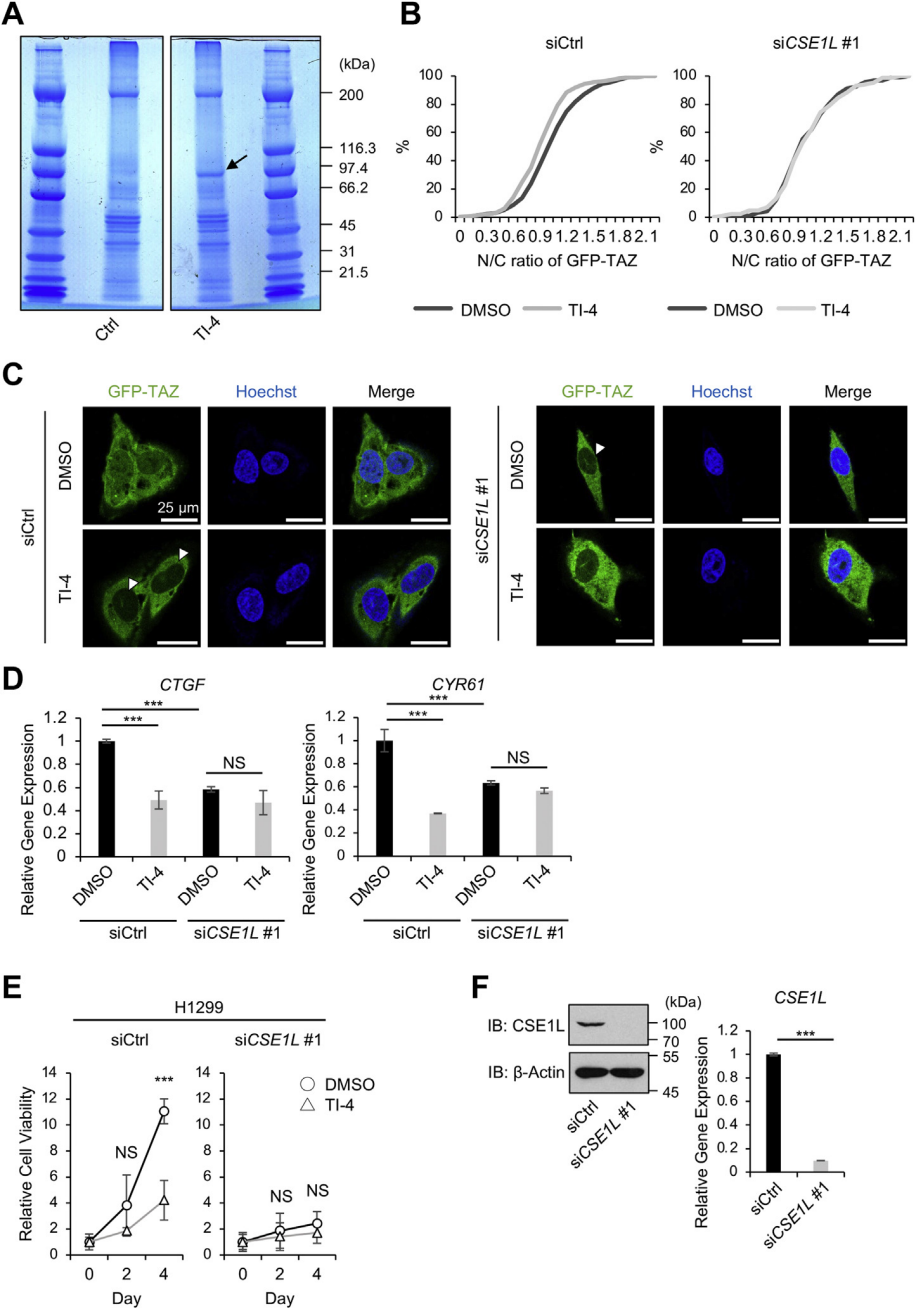
**CSE1L is a putative target of TI-4.***A*, the cell lysates of U87MG cells were incubated on either control or TI-4-conjugated beads as described in Experimental procedures. The *arrow* indicates the protein that specifically binds to TI-4-conjugated beads. *B* and *C*, U2OS-GFP-TAZ cells were plated at 1.5 × 10^5^ cells/well in a 6-well plate and transfected with control siRNA or *CSE1L* siRNA. Forty-eight hours later, the cells were replated at 3 × 10^3^ cells/well in a 96-well plate and cultured with or without 10 μM TI-4 for 48 h. The cells were fixed, and the nuclei were visualized with Hoechst 33342. GFP signals inside and outside the nucleus were measured, and the ratio of the nuclear GFP over the cytoplasmic GFP (N/C ratio) was calculated. In panel *B*, the distributions of N/C ratio in 300 cells are depicted as cumulative curves. In the CSE1L-negative background, TI-4 failed to shift the distribution. In panel *C*, TI-4 and *CSE1L* silencing reduced the nuclear GFP-TAZ (*arrowheads*). *D*, H1299 cells were plated at 1.5 × 10^5^ cells/well in a 6-well plate and transfected with control siRNA or *CSE1L* siRNA. Twenty-four hours after transfection, TI-4 was added to a final concentration of 10 μM, and the cells were further cultured for 48 h. Total RNA was extracted, and quantitative RT-PCR was performed to measure *CTGF* and *CYR61* mRNAs. Data are shown as the mean ± SD. ∗∗∗*p* < 0.001. *E*, H1299 cells were plated at 1.5 × 10^5^ cells/well in a 6-well plate and transfected with control siRNA or *CSE1L* siRNA. Twenty-four hours later, the cells were plated at 3000 cells/well in a 96-well plate. Twenty-four hours later, the cells were treated with DMSO or 10 μM TI-4 and cultured for 48 h and 96 h. The MTT assay was performed. Data are shown as the mean ± SD. ∗∗∗*p* < 0.001. *F*, the efficiency of *CSE1L* silencing was confirmed by immunoblotting and quantitative RT-PCR. The experiments were repeated twice for those mentioned in panel *A* and three times for those mentioned in panels *B*–*E*. CSE1L, chromosomal segregation 1 like; DMSO, dimethyl sulfoxide; GFP-TAZ, GFP-tagged TAZ; MTT, 3-[4,5-dimethylthiazol-2-yl]-2,5-diphenyl tetrazolium bromide; n.s., not significant; TI-4, TAZ inhibitor 4.

As TI-4 did not affect the phosphorylation state of TAZ but recruited TAZ into the cytoplasm, we speculated that TI-4 regulates TAZ through an unknown mechanism, which is distinct from the canonical Hippo pathway. With this in mind, we attempted to identify a target molecule of TI-4. We immobilized TI-4 onto Sepharose beads through a photoaffinity linker, charged the cell lysates of U87MG cells, and identified proteins that specifically bound to the beads (Fig. 3*A*). Although several protein bands were detected on control beads (Fig. 3*A*, Ctrl), the protein with approximately 97 kDa was detected only on TI-4-affinity beads (Fig. 3*A*, TI-4, an arrow). The protein was subjected to MS analysis and identified peptides derived from CSE1L (Fig. S2).

### *CSE1L* silencing attenuates the effect of TI-4

Next, we applied TI-4 to U2OS-GFP-TAZ cells after *CSE1L* silencing and examined whether CSE1L is required for TI-4 to recruit TAZ into the cytoplasm. *CSE1L* silencing abolished the effect of TI-4 (Fig. 3*B*). In the immunofluorescence, TI-4 decreased GFP-TAZ in the nucleus (Fig. 3*C*, siCtrl, arrowheads). *CSE1L* silencing itself reduced the nuclear GFP-TAZ (Fig. 3*C*, si*CSE1L* #1, an arrowhead), and TI-4 did not show any additional effect. We also examined in H1299 cells whether *CSE1L* silencing blocked the effect of TI-4 on the expression of *CTGF* and *CYR61*. *CSE1L* silencing itself suppressed the expression of *CTGF* and *CYR61* in HEK293FT cells (Fig. 3*D*, the first and third bars), while TI-4 did not decrease the expression of *CTGF* and *CYR61* in the *CSE1L*-negative background (Fig. 3*D*, the third and fourth bars). Furthermore, *CSE1L* silencing suppressed the cell viability of H1299 cells in MTT assay (Fig. 3*E*), whereas TI-4 did not show any additional effect in the *CSE1L*-negative background (Fig. 3*E*, si*CSE1L* #1). These results support that TI-4 requires CSE1L to inhibit TAZ/TEAD target genes and to show anticancer effect.

### CSE1L is involved in the regulation of the subcellular distribution of TAZ but has no effect on TAZ phosphorylation

**Figure 4:**
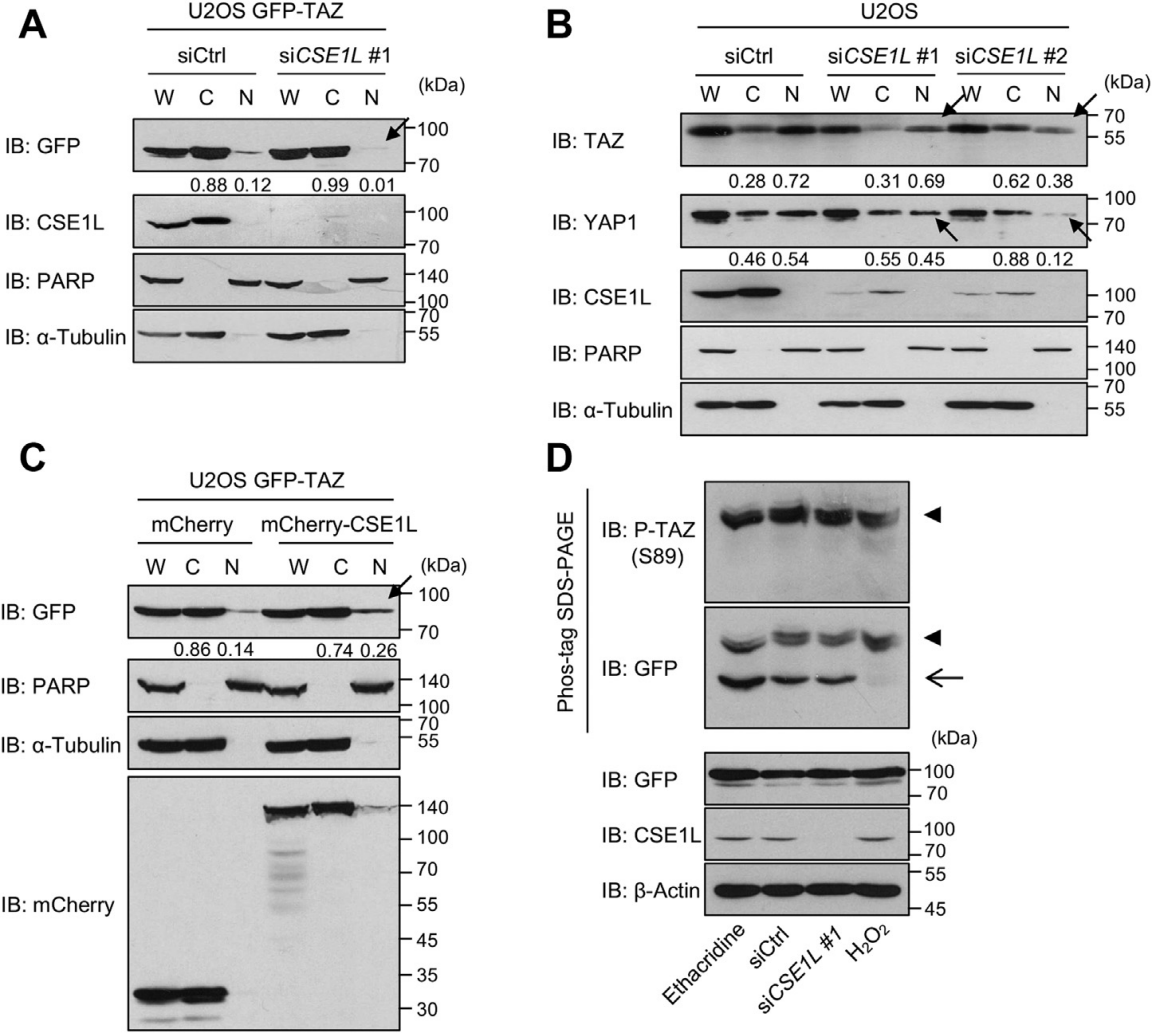
**The effect of CSE1L on the subcellular distribution of TAZ.***A* and *B*, U2OS-GFP-TAZ or U2OS cells were plated at 4.0 × 10^5^ cells/6-cm dish and transfected with control siRNA or *CSE1L*-siRNA. Seventy-two hours later, the subcellular fractionation was performed. PARP and α-tubulin were used as nuclear (N) and cytoplasmic (C) markers, respectively. The nuclear GFP-TAZ or endogenous TAZ was reduced by *CSE1L* silencing (*arrows*). Endogenous YAP1 was also decreased by *CSE1L* silencing (*arrows*). *C*, U2OS-GFP-TAZ cells were plated at 4 × 10^5^ cells/6-cm dish. Twenty-four hours later, the cells were transfected with control pCIneomCherry or pCIneomCherry-CSE1L. Forty-eight hours later, the subcellular fractionation was performed. The nuclear GFP-TAZ was increased by CSE1L expression (an *arrow*). The numbers under the immunoblottings indicate the ratio of cytoplasmic and nuclear TAZ in the indicated image. Other two experiments showed the similar results as shown in Fig. S3*A*. *D*, U2OS-GFP-TAZ cells were plated at 1.5 × 10^5^ cells/well in a 6-well plate and transfected with control siRNA or *CSE1L* siRNA. The whole-cell lysates were run on Phos-tag gels and immunoblotted with the indicated antibodies. As the control, the cells were treated with 10 μM ethacridine for 24 h and with 1 mM H_2_O_2_ for 30 min. Upper bands were detected with anti-phospho-TAZ (S89) and anti-GFP antibodies (*arrowheads*), while the lower band was detected only with anti-GFP antibody. Ethacridine increased unphosphorylated TAZ, whereas H_2_O_2_ reduced it (an *arrow*). The experiments were performed three times. CSE1L, chromosomal segregation 1 like; GFP-TAZ, GFP-tagged TAZ; PARP, poly (adp-ribose) polymerase; TAZ, transcriptional coactivator with PDZ-binding motif.

We next examined whether and how CSE1L affects the subcellular distribution of TAZ. *CSE1L* silencing reduced the nuclear GFP-TAZ in U2OS-GFP-TAZ cells and the endogenous nuclear TAZ in U2OS cells (Fig. 4, *A* and *B*, arrows). Conversely, CSE1L overexpression increased the nuclear TAZ (Fig. 4*C*, an arrow). *CSE1L* silencing did not increase unphosphorylated TAZ (Fig. 4*D*, an arrow). Furthermore, we knocked down *CSE1L* in U2OS cells expressing GFP-TAZ S89A (Fig. S1*B*). *CSE1L* silencing shifted TAZ S89A mutant to the cytoplasm. These findings support that the effect of CSE1L on the subcellular localization of TAZ does not depend on the phosphorylation state of TAZ.

### *CSE1L* silencing delays the recovery of nuclear GFP-TAZ after photobleaching

**Figure 5:**
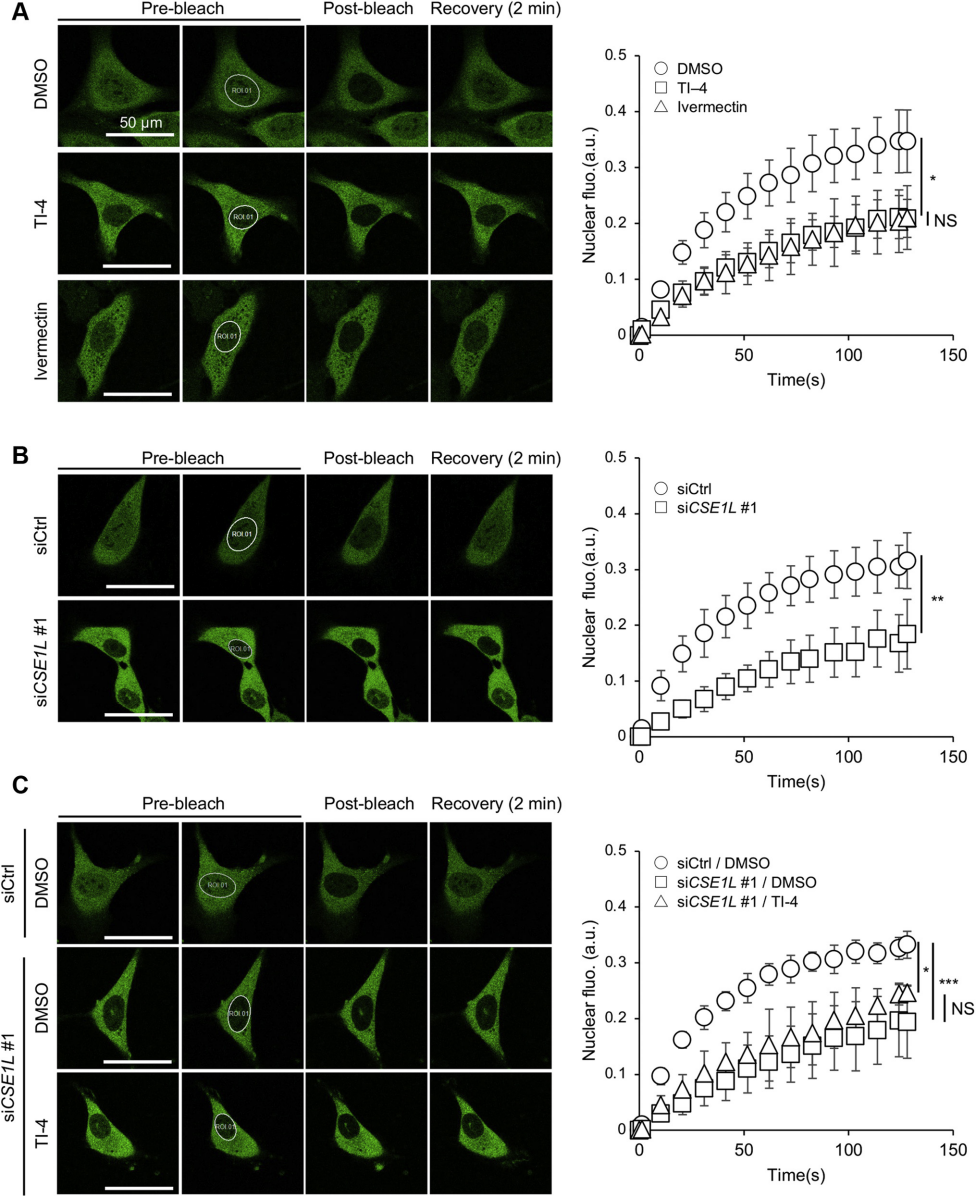
***CSE1L* silencing delays the recovery of nuclear GFP-TAZ after photobleaching.***A*, the effect of TI-4 and ivermectin. *B*, the effect of *CSE1L* silencing. *C,* the effect of TI-4 in the CSE1L-negative background. Representative images of FRAP experiments are demonstrated on the *left*. The bleached areas are demarcated with *white lines*. Fluorescent recovery rates were calculated as described in Experimental procedures. About 5~10 cells for each condition were measured for each experiment. Three independent experiments were performed. The scale bars represent 50 μm. ∗*p* < 0.05; ∗∗*p* < 0.01; ∗∗∗*p* < 0.001. CSE1L, chromosomal segregation 1 like; GFP-TAZ, GFP-tagged TAZ; FRAP, fluorescence recovery after photobleaching; NS, not significant.

We next performed fluorescence recovery after photobleaching (FRAP) assay. The nuclear GFP was photobleached in U2OS-GFP-TAZ cells. TI-4 delayed the recovery of the GFP signal in the nucleus (Fig. 5*A*, squares). Ivermectin, which was identified as the TAZ inhibitor in the drug screening and is thought to be an importin inhibitor, showed a similar effect (18) (Fig. 5*A*, triangles). As *CSE1L* silencing decreased nuclear GFP-TAZ, the basal intensity of the nuclear GFP signal before photobleaching was lower in CSE1L-depleted cells than in control cells. Even so, *CSE1L* silencing delayed the recovery (Fig. 5*B*). TI-4 did not show additional effect after *CSE1L* silencing (Fig. 5*C*).

### TI-4 strengthens the interaction between CSE1L and importin α5 and weakens the interaction between TAZ and importin α5

**Figure 6:**
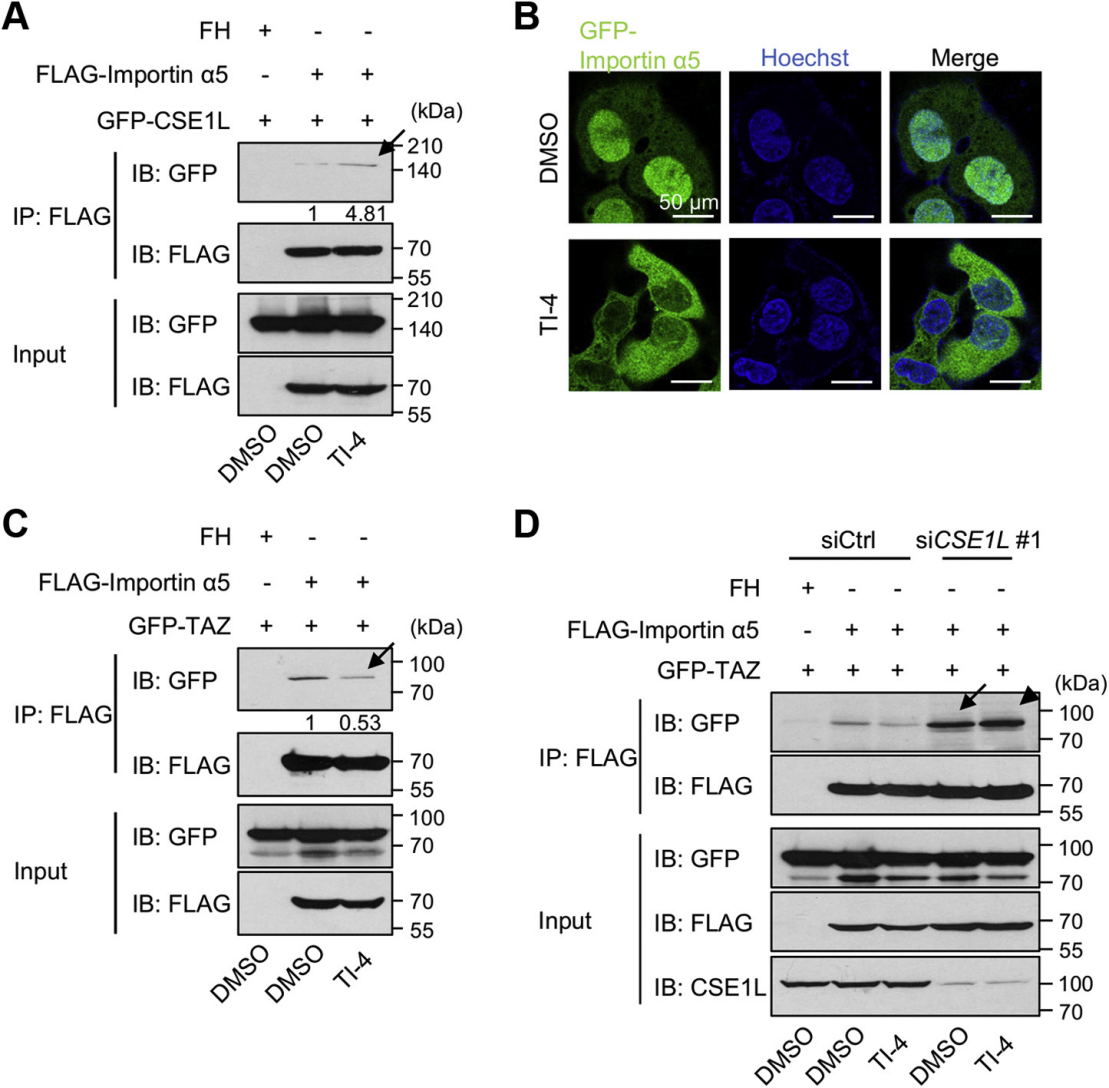
**TI-4 strengthens the interaction between CSE1L and importin α5 and weakens the interaction between TAZ and importin α5.***A* and *C*, HEK293FT cells were plated at 8 × 10^5^ cells/well in a 6-well plate. Sixteen hours later, cells were transfected with pCIneoFH-importin α5, pCIneoGFP-CSE1L, and pCIneoGFP-TAZ as indicated. Twenty-four hours later, the cells were treated with DMSO or 10 μM TI-4 and cultured for 48 h. The immunoprecipitation was performed with anti-DYKDDDDK-beads. *A*, CSE1L was coimmunoprecipitated with importin α5 under TI-4 treatment (an *arrow*). *C*, TI-4 treatment reduced the amount of coimmunoprecipitated TAZ with importin α5 (an *arrow*). *B*, U2OS-GFP-importin α5 cells were plated at 1 × 10^5^ cells/dish in a 35-mm dish, and 24 h later, treated with DMSO or 10 μM TI-4. Forty-eight hours later, the cells were fixed, and the nuclei were visualized with Hoechst 33342. TI-4 reduced the nuclear GFP-importin α5. *D*, HEK293FT cells were plated at 1 × 10^6^/dish in a 6-cm dish and transfected with control siRNA or *CSE1L* siRNA. Twenty-four hours later, the cells were replated at 8 × 10^5^ cells/well in a 6-well plate. Twenty-four hours later, the cells were transfected with pCIneoFH-importin α5 and pCIneoGFP-TAZ, cultured for 24 h, and then treated with DMSO or 10 μM TI-4. Forty-eight hours later, the immunoprecipitation was performed with anti-DYKDDDDK-tag beads. *CSE1L* silencing strengthened the interaction between importin α5 and TAZ (an *arrow*). Under this condition, TI-4 failed to inhibit the interaction between importin α5 and TAZ (an *arrowhead*). The experiments were performed three times. The numbers under the immunoblottings indicate the signal intensity of GFP-CSE1L and GFP-TAZ in the indicated image. Other two experiments showed the similar results as shown in Fig. S3, *B* and *C*. CSE1L, chromosomal segregation 1 like; DMSO, dimethyl sulfoxide; GFP-TAZ, GFP-tagged TAZ; TAZ, transcriptional coactivator with PDZ-binding motif; TI-4, TAZ inhibitor 4.

We investigated into the molecular mechanism, by which TI-4 delays the nuclear incorporation of TAZ. The alias of CSE1L is exportin-2. Importin α has a nuclear localization signal (NLS)-binding site, forms a complex with NLS-harboring proteins and importin β, and incorporates NLS-harboring proteins into the nucleus (19). In the nucleus, the complex is dissociated. Then CSE1L binds to the released importin α and exports it into the cytoplasm (11, 12). Thus, CSE1L contributes to the recycling of importin α. Recently, Kofler *et al.* (20) have revealed that TAZ has a new class NLS motif in the C-terminal region and a nuclear efflux signal in the N-terminal region. We tested whether TAZ can interact with importin α. We coexpressed GFP-TAZ with various FLAG-tagged importin α proteins in HEK293FT cells and performed the coimmunoprecipitation experiment and detected the interaction between TAZ and importin α proteins (Fig. S4*A*). We also immunoprecipitated TAZ from HEK293FT cells and detected the interaction between endogenous TAZ and importin α5 (Fig. S4*B*) and focused on importin α5 in this study. First, we confirmed that CSE1L is involved in the export of importin α5 in U2OS cells. We knocked down *CSE1L* in U2OS cells expressing GFP-importin α5. *CSE1L* silencing increased nuclear GFP-importin α5 in immunofluorescence and the subcellular fractionation (Fig. S5, an arrow). We subsequently examined whether and how TI-4 affects the interaction between CSE1L and importin α5. The interaction between CSE1L and importin α5 was hardly detected at the basal state, but TI-4 made it detectable (Fig. 6*A*, an arrow). We next examined whether and how TI-4 influenced the subcellular localization of importin α5. Under the treatment of TI-4, the nuclear importin α5 was reduced (Fig. 6*B*). These findings suggest that importin α5 remains to bind to CSE1L and is not incorporated into the nucleus under the treatment with TI-4. CSE1L binds to the C-terminal region of importin α and interferes with the interaction between the NLS and armadillo repeats (12). Hence, importin α does not bind to an NLS-harboring protein, until it is released from CSE1L. Considering the possibility that CSE1L competes with TAZ for the binding to importin α, we examined the effect of TI-4 on the interaction between TAZ and importin α5. TI-4 reduced the interaction between importin α5 and TAZ (Fig. 6*C*, an arrow). In contrast, *CSE1L* silencing remarkably augmented the interaction (Fig. 6*D*, an arrow). TI-4 did not inhibit the interaction between TAZ and importin α5 in the CSE1L-negative background (Fig. 6*D*, an arrowhead). These findings suggest that TI-4 blocks the dissociation of importin α5 from CSE1L in the cytoplasm and eventually inhibits the complex formation including importin α5 and TAZ.

### CSE1L regulates the nuclear localization of YAP1

CSE1L is involved in the recycling of importin α (11). CSE1L is required for the nuclear import of nuclear proteins including histone deacetylases (21). Therefore, we considered that CSE1L may regulate the subcellular distribution of other nuclear proteins. *CSE1L* silencing decreased the nuclear endogenous YAP1 in U2OS cells (Fig. 4*B*). We used U2OS cells expressing GFP-YAP1 and conducted FRAP assay (Fig. S6*A*). In FRAP experiment, *CSE1L* silencing delayed the recovery of the nuclear GFP-YAP1. However, when we immunoblotted the samples of subcellular fractionation, *CSE1L* silencing did not affect the nuclear localization of endogenous β-catenin and FoxO3a (Fig. S6*B*). Thus, although the underlying mechanism is unknown, CSE1L is involved in the regulation of the limited population of nuclear proteins.

### CSE1L and TAZ promote cell invasiveness, motility, and colony formation in cancer cells

**Figure 7:**
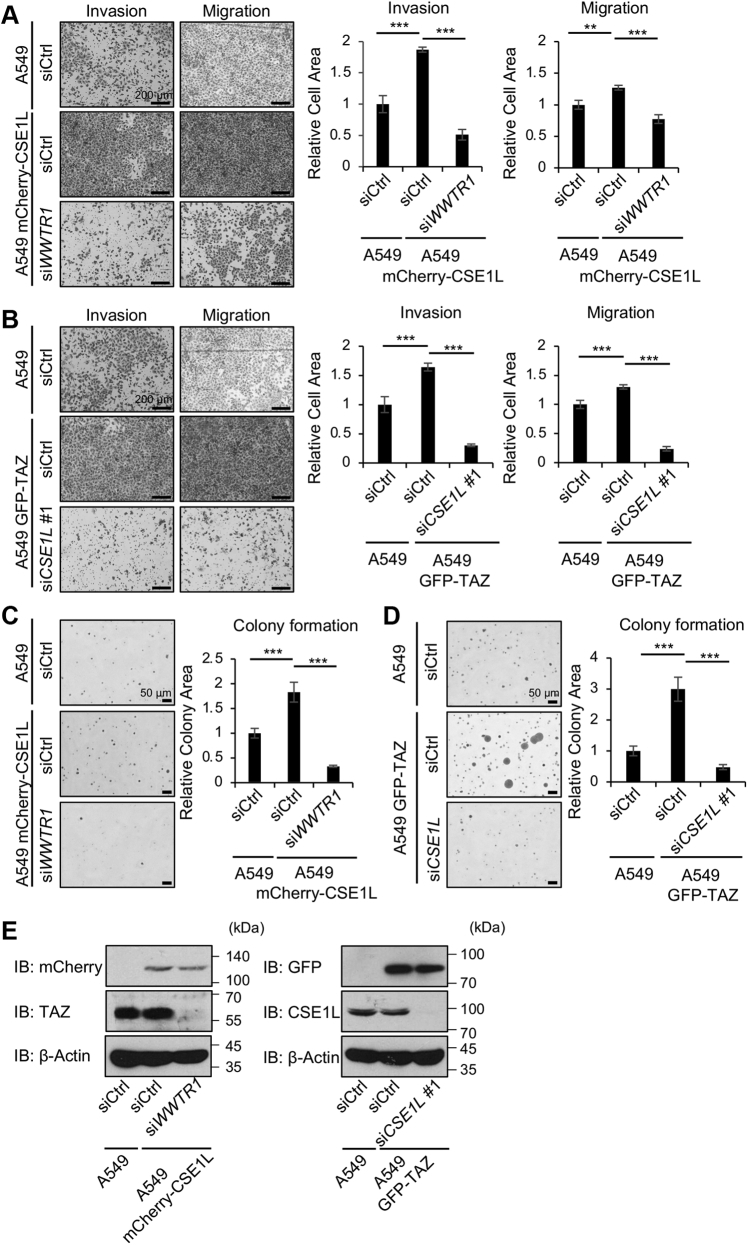
**CSE1L and TAZ co-operatively promote cell invasiveness, motility, and colony formation in A549 cells.** A549, A549-mCherry-CSE1L, and A549-GFP-TAZ cells were plated at 1.5 × 10^5^ cells/well in a 6-well plate and transfected with control siRNA, *WWTR1* siRNA, or *CSE1L* siRNA as indicated. In panels *A* and *B*, 24 h later, cells were transferred to the serum-free medium. Twenty-four hours later, cells were replated at 4 × 10^4^ cells/insert in 8.0-μm Transparent PET Membrane Insert, and invasion and migration assays were performed as described in Experimental procedures. Forty hours later, cells were stained by crystal violet. The migrating or invading cells were observed under the microscope in three independent fields for each condition and quantified using ImageJ. In panels *C* and *D*, 24 h later, cells were replated 3000 cells/well in 12-well plate for the soft agar assay as described in Experimental procedures. Two weeks later, three independent images for each sample were obtained with a microscope BZ-X700 (Keyence) and analyzed by BZ-H3C Hybrid Cell Count Module. Data are shown as the mean ± SD. ∗∗*p* < 0.01; and ∗∗∗*p* < 0.001. The experiments were performed three times. *E*, the cells were immunoblotted to confirm the expression of mCherry-CSE1L and GFP-TAZ and to show the efficiency of *WWTR1* silencing and *CSE1L* silencing. CSE1L, chromosomal segregation 1 like; GFP-TAZ, GFP-tagged TAZ; TAZ, transcriptional coactivator with PDZ-binding motif.

In human cancers, CSE1L confers malignant properties to cancer cells and is regarded as a poor prognostic biomarker (22). We analyzed the public data of cancer patients with PrognoScan (http://gibk21.bse.kyutech.ac.jp/PrognoScan/index.html) (Fig. S7). Patients with high expression of *CSE1L* exhibited shorter survival in breast cancer (GSE4922, GSE11121), brain tumor (GSE4271), liposarcoma (GSE30929), lung cancer (GSE13213), and ovarian cancer (GSE9891). We raised a question whether and how TAZ contributes to CSE1L-mediated enhancement of malignancy. CSE1L promoted cell migration and invasiveness in A549 cells in transwell assays, whereas *WWTR1* silencing attenuated the effect of CSE1L (Fig. 7*A*). Likewise, CSE1L promoted colony formation in the soft agar assay, but *WWTR1* silencing blocked it (Fig. 7*C*). Conversely, we also observed that *CSE1L* silencing reduced TAZ-mediated malignant transformation in A549 cells (Fig. 7, *B* and *D*). We performed the same experiments using U87MG cells and obtained similar results (Fig. S8). Hence, it is reasoned that CSE1L increases TAZ in the nucleus and induces malignant transformation in cancer cells. On the other hand, it is likely that TAZ, even when expressed, fails to enhance malignancy in cancer cells lacking CSE1L.

### High expression of CSE1L correlates with high expression of nuclear TAZ in human cancers

Finally, we wanted to know whether and how CSE1L expression correlates with TAZ expression in cancer patients. To this aim, we used human cancer microarray, evaluated CSE1L expression by using a semiquantitative scale of immunoreactive score and examined whether TAZ was detected in the nucleus in human cancers with high levels of CSE1L (Fig. S9). Of 45 lung adenocarcinoma and 16 lung squamous cell carcinoma cases, 33 and 12 cases were classified as CSE1L-high cases, respectively. All CSE1L-high cases express high levels of nuclear TAZ (Table 1). In contrast, nuclear TAZ expression was high in none of CSE1L-low adenocarcinoma and squamous cell carcinoma cases. In gastric and esophageal cancers, most cases did not express high levels of CSE1L (18% for gastric cancers and 15% for esophageal cancers). Nevertheless, CSE1L-high cancers tended to express TAZ at the high level in these cancers. Thus, high expression of CSE1L correlates with high expression of nuclear TAZ.

**Table 1:** Expression of CSE1L and nuclear TAZ in human cancers

Nuclear TAZ expression	High	Low	Sum
(A) Lung cancer			
(ⅰ)Adenocarcinoma			
CSE1L expression			
High	33	4	37
Low	0	8	8
Sum	33	12	45
(ⅱ) Squamous cell carcinoma			
CSE1L expression			
High	12	1	13
Low	0	3	3
Sum	12	4	16
(B) Gastric cancer			
CSE1L expression			
High	9	0	9
Low	20	21	41
Sum	29	41	50
(C) Esophageal cancer			
CSE1L expression			
High	4	3	7
Low	1	39	40
Sum	5	42	47

(A) (i) Chi square, 26.757, *p* = 0.0000002; (ii) chi square, 11.077, *p* = 0.0008741.

(B) Chi square, 7.95, *p* = 0.005; (C) chi-square, 18.71, *p* = 0.00002.

## Discussion

In this article, we reported the novel TAZ inhibitor, TI-4. TI-4 shifts TAZ to the cytoplasm from the nucleus independent of TAZ phosphorylation, which suggests that the target of TI-4 is not a component of the canonical Hippo pathway. We identified CSE1L as its target.

**Figure 8:**
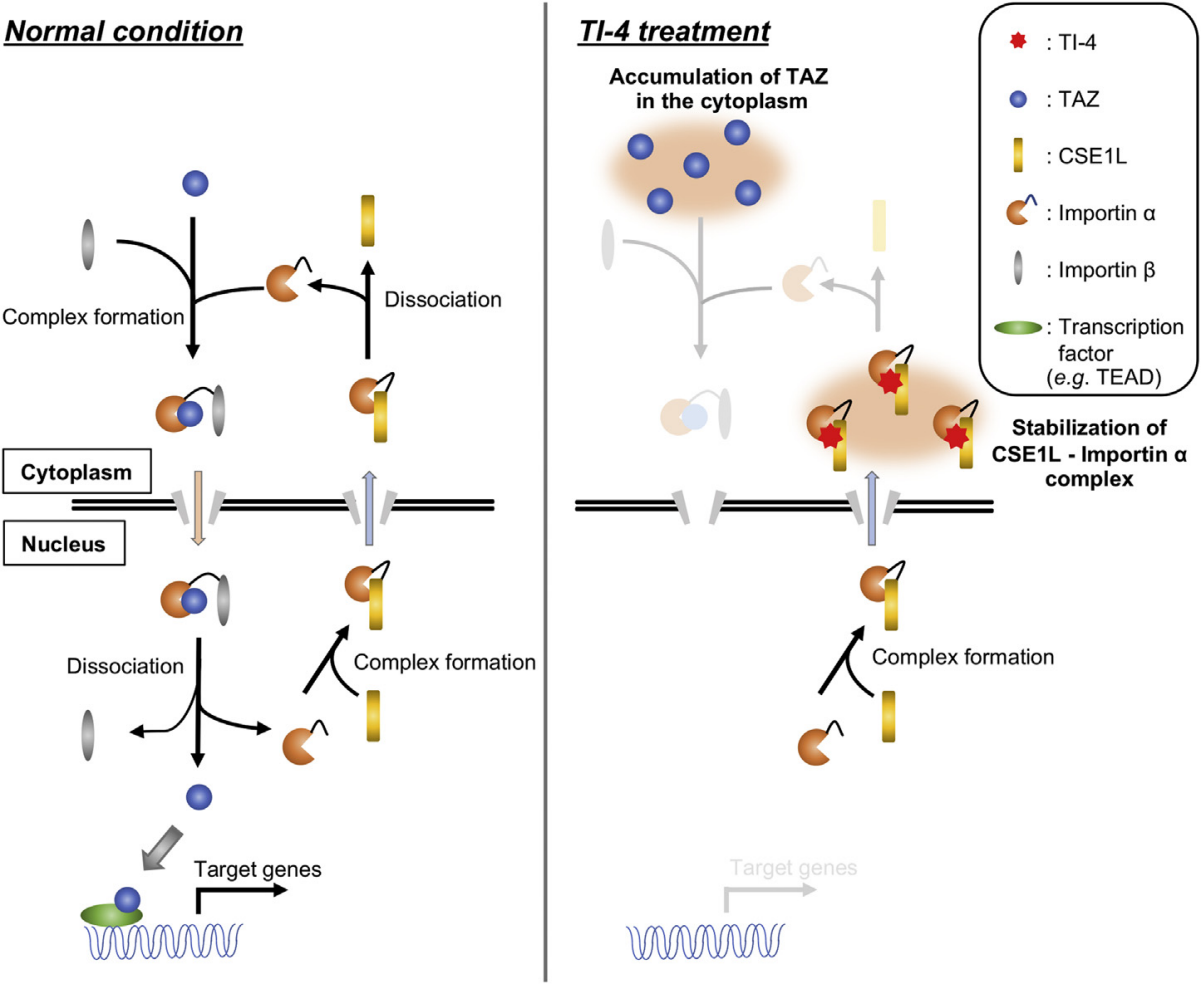
**The putative mechanism by which TI-4 inhibits the nuclear incorporation of TAZ.** TAZ (*blue symbols*) forms a complex with importin α (*orange symbols*) and importin β (*gray symbols*) and is incorporated into the nucleus. The complex is dissociated in the nucleus. CSE1L (*yellow symbols*) binds to the released importin α and recruits it to the cytoplasm. Importin α is dissociated from CSE1L in the cytoplasm and is used for the next cycle to import TAZ into the nucleus. TI-4 blocks the dissociation of importin α from CSE1L. Therefore, importin α is accumulated in the cytoplasm, and TAZ fails to bind to importin α, so that TAZ cannot enter the nucleus. CSE1L, chromosomal segregation 1 like; TAZ, transcriptional coactivator with PDZ-binding motif; TI-4, TAZ inhibitor 4.

CSE1L is a mammalian homologue of yeast Cse1, which associates with chromatin, and was identified as the gene that made MCF-7 cells resistant to immunotoxin (8). CSE1L was also identified as an importin α−binding protein (11, 12). To maintain the nuclear transport, importin α must return from the nucleus to the cytoplasm. CSE1L is involved in the nuclear export of importin α. A previous study revealed that the nuclear import of histone deacetylases is suppressed by CSE1L depletion (21). Therefore, we suspected that CSE1L is also required for the nuclear import of TAZ. *CSE1L* silencing indeed decreased the nuclear TAZ, whereas CSE1L increased it. Despite the importance of the subcellular localization in the regulation of YAP1 and TAZ, our knowledge regarding the nuclear cytoplasmic transport of these proteins is yet limited. In *Drosophila melanogaster*, importin α1 binds to the N-terminal 55 amino acids of Yorkie, a homologue of YAP1/TAZ, and drives it into the nucleus (23). Although the N-terminal sequence of TAZ is different from that of Yorkie and TAZ has the NLS not in the N-terminal region but in the C-terminal region (20), we detected the coimmunoprecipitation of TAZ and importin α5. As CSE1L interferes with the binding of NLS to importin α, CSE1L is supposed to block the interaction between TAZ and importin α. Consistently, *CSE1L* silencing enhanced the interaction between TAZ and importin α5. TI-4 strengthened the interaction between CSE1L and importin α and weakened the interaction between TAZ and importin α depending on CSE1L. All these findings support the model that CSE1L facilitates TAZ nuclear incorporation through recycling importin α and that TI-4 prevents the formation of the complex including TAZ and importin α to block the nuclear entry of TAZ (Fig. 8).

In human cancers, high expression of CSE1L is associated with a poor prognosis. CSE1L enhances drug resistance, invasion, and metastasis (13, 14, 24). Serum CSE1L is regarded as a prognostic marker of cancers (25, 26). The mechanism, by which CSE1L confers malignant properties to cancer cells, is not yet fully understood. Various explanations are proposed. CSE1L is associated with a set of p53 target genes and suppresses them (27). CSE1L mediates the silencing of methylated genes (21). CSE1L suppresses tumor suppressor *RASSF1* gene expression (28). CSE1L reduces the nuclear accumulation of RAD51 and is involved in the regulation of DNA repair (29). CSE1L is implicated in the activation of Ras/ERK, cAMP/PKA, and microphthalmia-associated transcription factor signals (30). We observed that TAZ is detected in the nucleus in CSE1L-expressing cancer cells and that *WWTR1* silencing antagonizes the effect of CSE1L in cancer cells. Conversely, *CSE1L* silencing attenuates the effect of TAZ. These findings suggest that TAZ functions downstream of CSE1L, while CSE1L augments the effect of TAZ by shifting TAZ into the nucleus.

One intriguing question is whether TI-4 is effective for epithelioid hemangioendothelioma (EHE) (31). EHE is a soft-tissue sarcoma caused by the fusion of TAZ and calmodulin-binding transcription activator 1 (CAMTA1). TAZ–CAMTA1 is released from the regulation by the Hippo pathway and constitutively activates TAZ-mediated transcription. If the nuclear import of TAZ–CAMTA1 depends on importin α, TI-4 may be useful to control EHE.

In conclusion, we revealed through the analysis of a new TAZ inhibitor that CSE1L accumulates TAZ in the nucleus and induces malignant transformation in human cancers.

## Experimental procedures

### DNA constructions and virus production

pCIneoFLAG-His6 (FH), pCIneoGFP-TAZ, pCIneoGFP-YAP1, pLL3.7-EGFPC2-TAZ, pLL3.7-EGFPC2-TAZ S89A, pLL3.7-EGFP2-YAP1 were previously described (7, 32). 8xGTIIC-δ51LucII luciferase reporter, pcDNA-Myc-His6-CSE1L, pcDNA FLAG-Importin α1, α3, and α4 are generous gifts of Hiroshi Sasaki (Osaka University), Satoshi Tashiro (Hiroshima University), and Koh Nakayama (Tokyo Medical and Dental University) (29, 33). PCR was performed with primers (H3691, 5′-acgcgtatggaactcagcgatgcaaatctgcaa-3′ and H3692, 5′-agtcgacttaaagcagtgtcacactggctgcctg-3′) on pcDNA-Myc-His6-CSE1L. The PCR product was digested with MluI/Sall and ligated into the same sites of pCIneomCherry to generate pCIneomCherry-CSE1L. NheI/Sall fragment from pCIneomCherry-CSE1L was ligated into NheI/XhoI sites of pLL3.7-ires-puro to generate pLL3.7-mCherry-CSE1L-ires-puro. PCR was performed with primers (H3753, 5′-acgcgtgaattcatgcccaggaaaagagaa-3′ and H3754, 5′-actagtcgacttcaagaaaccttccatagg-3′) on human kidney and lung cDNAs (Clontech). The PCR product was ligated into MluI/SalI sites of pCIneoFH and pCIneoEGFPC2 to generate pCIneoFH- and pCIneoEGFPC2-importin α5. NheI/Sall fragment from pCIneoEGFPC2-importin α5 was ligated into NheI/XhoI sites of pLL3.7-ires-puro to generate pLL3.7-GFP-importin α5-ires-puro.

### Antibodies and reagents

Rabbit anti-phospho-TAZ antibody was previously described (34). Other antibodies and the reagents used in this study are as follows: mouse monoclonal anti-GFP (sc-9996), mouse monoclonal anti-poly (adp-ribose) polymerase (sc-8007), mouse monoclonal anti-CAS (H-2) (sc-271537), mouse monoclonal karyopherin α1 (187.1) (sc-101292) (Santa Cruz Biotechnology); mouse monoclonal anti-TAZ (560235) (BD Biosciences); rabbit polyclonal anti-α-tubulin (PM054) and rabbit polyclonal anti-β-actin (PM053) (Medical and Biological Laboratories Co Ltd); anti-DYKDDDDK-tag (014-22383) and anti-DYKDDDDK-beads (016-22784) (Wako Pure Chemical Industries); rabbit polyclonal anti-mCherry (GeneTex); rabbit monoclonal FoxO3a (75D8)(2497) and rabbit monoclonal YAP1(D8H1X)XP (14074) (Cell Signaling Technology); rabbit polyclonal importin α5 (18137-1-AP) and rabbit polyclonal TAZ (23306-1-AP) (Proteintech Group, Inc); mouse monoclonal β-catenin (13-8400) (Zymed Laboratory Inc); Protein G Sepharose 4 Fast Flow (17-0618-01) (GE Healthcare BioSciences); Rabbit IgG Isotype Control (31235) (Thermo Fisher Scientific); and TI-4 (2,2-dichloro-N-(4-nitrophenyl)-3-phenylcyclopropanecarboxsmide) (STK825627; Vitas-M Chemical Limited).

### Cell cultures and gene introduction

HEK293FT, U2OS, H1299, A549, and U87MG cells were cultured in Dulbecco’s modified Eagle's medium (DMEM) containing 10% (v/v) fetal bovine serum (FBS) and 10 mM Hepes-NaOH, pH 7.4, under 5% CO_2_ at 37 °C. Cell authentication was performed by using short-tandem repeat profile (GenePrint 10 System) (Promega). *Mycoplasma* infection was periodically checked by using e-Myco *Mycoplasma* PCR Detection Kit (ver.2.0) (#25235, iNtRON Biotechnology). When infection was detected, *Mycoplasma* elimination was performed by Plasmocin treatment (#ant-mpt-1, InvivoGen). DNA transfection was performed with Lipofectamine 2000 (Life Technologies). Stable transformant cells were prepared by using lentivirus vectors, pLL3.7-EGFPC2-TAZ, pLL3.7-EGFPC2-TAZ S89A, pLL3.7-EGFP2-YAP1, pLL3.7-GFP-importin α5, and pLL3.7-mCherry-CSE1L.

### Chemical compound screening

TI-4 was identified in the chemical compound screening that was previously reported (7). Briefly, U2OS-GFP-TAZ cells were plated at 3000 cells/well in 96-well plates. Cells were treated with 10 μM compounds for 16 h. The nucleus was visualized with Hoechst 33342. GFP signals inside and outside the nucleus were measured by ArrayScan VTI in 300 cells for each compound (Thermo Fisher Scientific). Robust Z-scores (|x-median(x)|/normalized interquartile range) were calculated. 86 compounds with a robust Z-score less than 3.0 were selected. 32 compounds were omitted because of the cytotoxicity. We evaluated by using TAZ/TEAD reporter assays in HEK293FT cells 33 compounds that reduced the nuclear TAZ to less than 90% in the second screening. We focused on five compounds (IBS000540, IBS008420, IBS001594, IBS015181 (renamed as TI-4 in this study), and IBS015625) and previously characterized three compounds (IBS000540, IBS001594, and IBS015625). IBS008420 later dropped out because of autoimmunofluorescence.

### Preparation of control and TI-4-affinity beads

TI-4-affinity column beads were prepared by the use of photoaffinity linker–coated Sepharose beads. Detailed protocol is available (35).

### Isolation of interacting molecules on TI-4-affinity beads

U87MG cells were plated at 3 × 10^6^ cells in a 10-cm dish. Twenty-four hours later, the cells were harvested from four dishes with PBS, transferred into one 1.5-ml microtube and centrifuged at 800*g* for 5 min at 4 °C. The cell pellets was resuspended in 1 ml of buffer A (20 mM Tris-HCl pH7.4, 5 mM MgCl_2_, 5 mM CaCl_2_, 1 mM EDTA, 1 mg/l (p-amidinophenyl)methanesulfonyl fluoride hydrochloride (APMSF), 1 mg/l leupeptin and 1 mg/l pepstatin A), incubated for 30 min on ice, then homogenized with 40 strokes in a glass homogenizer, and centrifuged at 100,000*g* for 20 min at 4 °C. The supernatant was incubated with 5 μl of the control beads for 1 h and centrifuged at 2300*g* for 1 min at 4 °C. The half aliquots were transferred into two 1.5-ml microtubes. Five microliter of either control or TI-4-conjugated beads was added to each tube, incubated for 24 h at 4 °C, and centrifuged at 2300*g* for 1 min. The beads were washed four times with buffer A and finally resuspended in 30 μl of the buffer. Fifteen microliter of the SDS sample buffer (180 mM Tris-HCl pH 6.8, 6% (w/v) SDS, 30% (v/v) glycerol, 0.06% (w/v) bromophenol blue, and 15% (v/v) β-mercaptoethanol) was added and boiled. Thirty microliter of each sample was charged and analyzed on SDS-PAGE.

### MS

The parameters of MS were summarized in Table S1. The immunoprecipitated samples separated by SDS-PAGE were stained with Coomassie brilliant blue (Quick Blue Staining Solution, #DS500, BioDynamics Laboratory Inc). Gel pieces were washed with 5% acetic acid and 50% methanol and then dehydrated with 66% acetonitrile and 17 mM NH_4_HCO_3._ The samples were incubated with 10 mM DTT at 60 °C for 1 h and blocked with 50 mM iodoacetamide at room temperature (RT) for 45 min, followed by digestion with 1 pmol of sequencing-grade trypsin (Promega). The peptides were injected into a nano-UHPLC system (Bruker Daltonics). Mass analysis was performed on a maXis-4G-CPR mass spectrometer equipped with a nano-ESI source. Magic C18AQ UHPLC Nano-Trap column (5-μm particle size, 200 Å pore diameter) (Biologica Co) and L-column ODS (0.1 × 150 mm., 3-μm particle size, Chemicals Evaluation and Research Institute) were used. The peptides were eluted from the column using a 10% (v/v) to 35% (v/v) acetonitrile gradient over 50 min. The eluted peptides were electrosprayed into the spectrometer. MS/MS spectra were acquired in a data-dependent mode.

### Quantitative RT-PCR

Quantitative RT-PCR analysis was performed by using SYBR Green (Roche) and ABI7500 Real-Time PCR system (Applied Biosystems). The used primers are human *GAPDH*, 5′-ccactcctccacctttgac-3′ and 5′-accctgttgctgtagcca-3′; human *CTGF*, 5′-ccaatgacaacgcctcctg-3′ and 5′-tggtgcagccagaaagctc-3′; human *CYR61*, 5′-agcctcgcatcctatacaacc-3′, and 5′-ttctttcacaaggcggcactc-3′; human *WWTR1*, 5′-tatcccagccaaatctcgtg-3′, and 5′-ttctgctggctcagggtact-3′; and human *CSE1L*, 5′-cggttcaaacacaatagcaagtg-3′ and 5′-ctgattcaggaggcatgtgct-3′.

### RNA interferences

RNA interferences were performed with Lipofectamine RNAiMAX (Thermo Fisher Scientific). The dsRNAs (Ambion) were as follows: s24787 for human *WWTR1*, s3590 for human *CSE1L*, and Silencer Select negative control No.2 #4390846 (Thermo Fisher Scientific); and sc-29908 CAS siRNA for human *CSE1L* (Santa Cruz Biotechnology). The validity of the knockdown was confirmed by quantitative RT-PCR or immunoblotting.

### Subcellular fractionation

U2OS or U2OS-GFP-TAZ cells were plated at 5 × 10^5^/dish in a 10-cm dish. Twenty-four hours later, cells were harvested by scraping in PBS and transferred into 1.5-ml microtubes. After centrifugation at 800*g* for 5 min, the pellets were resuspended five times by 200-μl yellow pipette in 200-μl extraction buffer (10 mM Hepes-NaOH pH7.4, 1.5 mM MgCl_2_, 10 mM KCl, 0.34 M sucrose, 10% (v/v) glycerol, 0.05% (v/v) NP-40, and 10 mg/l APMSF) and incubated for 5 min on ice. Seventy microliter of the mixture was saved as the whole cell lysate. The remaining mixture was centrifuged at 800*g* for 5 min, and the supernatant was saved as the cytoplasmic fraction. The pellet was washed once, resuspended in 130-μl extraction buffered, and used as the nuclear fraction.

### Immunoprecipitation

HEK293FT cells were plated at 8 × 10^5^ cells/well in a 6-well plate. Sixteen hours later, cells were transfected with various combinations of expression vectors. The total amount of DNA was adjusted to 2 μg for all the points. Twenty-four hours later, cells were treated with dimethyl sulfoxide or 10 μM TI-4, further cultured for 48 h, and then lysed in 500-μl lysis buffer (25 mM Tris-HCl, pH 7.4, 150 mM NaCl, 2 mM EDTA, 10 mM MgCl_2_, 1% (v/v) Triton X-100, 10% (v/v) glycerol, 10 mg/l APMSF, and 10 mg/l leupeptin). The supernatant was collected after centrifugation at 20,000*g* for 10 min. Forty microliter of the supernatant was stocked as the input, and 400 μl of the supernatant was used for the immunoprecipitation with 5-μl anti-DYKDDDDK-tag beads. To detect the coimmunoprecipitation of endogenous TAZ and importin α5, *CSE1L* was silenced in advance. HEK293FT cells were plated at 3 × 10^6^ cells/dish in a 10-cm dish and were transfected with *CSE1L* siRNA. Seventy-two hours later, the cells were lysed in the lysis buffer. The supernatant was collected after centrifugation at 20,000*g* for 10 min. Four hundred microliter of the supernatant was incubated with 1 μg of rabbit control IgG or TAZ polyclonal antibody for 2 h and then with 5-μl Protein G Sepharose 4 Fast Flow protein G beads. Proteins were detected in the inputs and the immunoprecipitates with appropriate antibodies.

### Phosphate-affinity SDS-PAGE

Phosphate-affinity SDS-PAGE was performed with Phos-tag acrylamide and polyvinylidene difluoride membranes (Millipore) as described previously (31). We used ethacridine and H_2_O_2_ as the control. Ethacridine dephosphorylates and activates TAZ (31). H_2_O_2_ activates mammalian Ste20-like kinases and phosphorylates TAZ.

### FRAP assay

The FRAP assay was performed by using a confocal laser-scanning microscope (LSM 510 Meta, Carl-Zeiss). U2OS-cells expressing GFP-TAZ or GFP-YAP1 were plated at 1 × 10^5^ cells/dish in a μ-Dish 35-mm (ib81156, NIPPON Genetics Co Ltd). Cells were stained with DRAQ5 (BioStatus Ltd) to visualize nuclei 30 min before observation. Selected cells were photobleached inside the nucleus by laser pulses at 488 nm (100% power, once capture) at 37 °C. Thereafter, fluorescence recovery was monitored for 130 s at 1.3-s intervals. The fluorescent recovery was evaluated according to the published method (36). Briefly, GFP signals were measured in the nucleus (N) and the whole cell (W) before (pre) and after the photobleaching (t). The cytoplasmic GFP (Cyto) signals were measured only before the photobleaching. The formula (F) to estimate the recovery was defined as follows.

ThenumeratorofF(t)=(N(t)−N(0))×Cyto(pre)×W(0)

ThedenominatorofF(t)=W(t)×W(pre)×W(t)

As photobleaching annihilated the nuclear GFP-TAZ and reduced the total amount of GFP-TAZ, W(t) was corrected by multiplying with W(pre)/Cyto(pre). W(0)/W(t) was a correction factor to compensate GFP-TAZ that was newly produced during observation.

### MTT and soft agar assays

MTT and soft agar assays were performed as described previously (7). Cells were plated 3000 cells/well in a 96-well plate for the MTT assay. The colorimetric assay was performed with thiazolyl blue tetrazolium bromide. Insoluble formazan was measured by iMark Microplate Absorbance Reader (Bio-Rad) at 595 nm. For the soft agar assay, 500 μl DMEM containing 10% (v/v) FBS, 0.5% (w/v) agarose, and 10 μM TI-4 was plated in one well of a 12-well plate. Then, 500-μl DMEM containing 3000 cells, 10% (v/v) FBS, 0.3% (w/v) agarose, and dimethyl sulfoxide or 10 μM TI-4 was overlaid and kept for 1 h at RT. After that, 250-μl DMEM containing 10% (v/v) FBS was further overlaid and cultured for 2 weeks. To evaluate the effect of the silencing of *CSE1L* or *WWTR1*, A549 and U87MG cells were transfected with either control siRNA or *WWTR1* siRNA. Twenty-four hours later, the cells were used for the soft agar assay. The images were obtained with a fluorescence microscope BZ-X700 (Keyence) and analyzed by BZ-H3C Hybrid Cell Count Module. The areas of cells were measured from three independent fields.

### Cell invasive assay

Cell migration and invasive assays were performed with 6.5-mm Transwell with 8.0-μm Transparent PET Membrane Insert (353097) (Corning Inc). The inserts were incubated with the fibronectin solution of 66 μg/ml at 4 °C for 24 h. The inserts used for invasion assay was further coated with 30-μg Matrigel in 100 μl of 10 mM Tris HCl, pH 8.0, containing 0.7% (w/v) NaCl. After centrifugation at 1000*g* for 10 min, the plate was incubated at 37 °C for 30 min, and 4 × 10^4^ A549 or U87MG cells were suspended in 100 μl DMEM, added to each plate, and incubated at 37 °C. Ten minutes later, 600-μl DMEM containing 10% FBS was added to the lower chamber. Forty hours later, the medium and Matrigel were removed by a cotton swab from the top of permeable support. The cells on the lower surface were fixed in 70% (v/v) ethanol and stained with 0.2% (w/v) crystal violet. The permeable supports were dried. The migrating or invading cells were observed under the microscope and quantified using ImageJ (https://imagej.net/).

### Immunohistochemistry of human cancers

This study was conducted according to the Declaration of Helsinki guidelines, and the study design of using human pathology archives has been approved by the Institutional Review Board of Hamamatsu University School of Medicine (20-011 for H. S.). Paraffin-embedded tissue samples from 235 cases of adenocarcinoma of the lung and 161 cases of squamous cell carcinoma of the lung who had undergone surgery at Hamamatsu University Hospital were used for the tissue microarray block. To explain in further detail, the tissue microarray block was prepared by transferring a cylinder of 3-mm-diameter tumor portion from each block containing resected tumor by using a microarrayer (KIN-1, Azumaya). The histopathological diagnosis of lung cancer was confirmed in Diagnostic Division of Hamamatsu University Hospital. This study was conducted with the approval of the Institutional Review Board of Hamamatsu University School of Medicine. The images were evaluated by two independent observers and were classified into high and low groups.

### Statistical analysis

Two samples were compared with the Student’s *t* test. ANOVA with Tukey’s test was used for the multiple comparison.

## Data availability

The mass spectrometry proteomics data have been deposited to the ProteomeXchange Consortium *via* the PRIDE partner repository with the dataset identifier PXD024194 and 10.6019/PXD024194.

## Supporting information

This article contains supporting information.

## Conflict of interest

The authors declare that they have no conflicts of interest with the contents of this article.
