# Supplementary material for: CSE1L promotes nuclear accumulation of transcriptional coactivator TAZ and enhances invasiveness of human cancer cells
Source: J Biol Chem. 2021 May 20;297(1):100803. doi: 10.1016/j.jbc.2021.100803 (PMC8209642; doi:10.1016/j.jbc.2021.100803)
Supplement: Supplemental Figures S1–S9 and Table S1 [file mmc1.pdf]

CSE1L promotes nuclear accumulation of transcriptional coactivator TAZ and enhances invasiveness of human cancer cells

Shunta Nagashima[1], Junichi Maruyama[1,*], Kaori Honda[2], Yasumitsu Kondoh[2], Hiroyuki Osada[2], Makiko Nawa[3], Ken-ichi Nakahama[4], Mari Ishigami-Yuasa[5], Hiroyuki Kagechika[5,6], Haruhiko Sugimura[7], Hiroaki Iwasa[1], Kyoko Arimoto-Matsuzaki[1], Hiroshi Nishina[8], Yutaka Hata[1,9,*]

[1]Department of Medical Biochemistry, Graduate School of Medical and Dental Sciences, Tokyo Medical and Dental University, Tokyo 113-8510, Japan

[2]Chemical Biology Research Group, RIKEN Center for Sustainable Resource Science, Saitama 351-0198, Japan.

[3]Laboratory of Cytometry and Proteome Research in Nanken-Kyoten and RCC, Tokyo Medical and Dental University, Tokyo 113-8510, Japan

[4]Department of Cellular Physiological Chemistry, Tokyo Medical and Dental University, Tokyo 113-8510, Japan

[5]Chemical Biology Screening Center, [6]Institute of Biomaterials and Bioengineering, Tokyo Medical and Dental University, Tokyo 101-0062, Japan

[7]Department of Tumor Pathology, Hamamatsu University School of Medicine, Hamamatsu 431-3192, Japan.

[8]Department of Developmental and Regenerative Biology, Medical Research Institute, Tokyo Medical and Dental University, Tokyo 113-8510, Japan

[9]Center for Brain Integration Research, Tokyo Medical and Dental University, Tokyo 113-8510, Japan

Running title: Implication of CSE1L in the nuclear import of TAZ

To whom correspondence should be addressed: Junichi Maruyama and Yutaka Hata, Department of Medical Biochemistry, Graduate School of Medical and Dental Sciences, Tokyo Medical and Dental University, Tokyo 113-8510, Japan

Tel: +81-3-5803-5164 Fax: +81-3-5803-0121

E-mail: jmaruyama.mbc@tmd.ac.jp and yuhammch@tmd.ac.jp

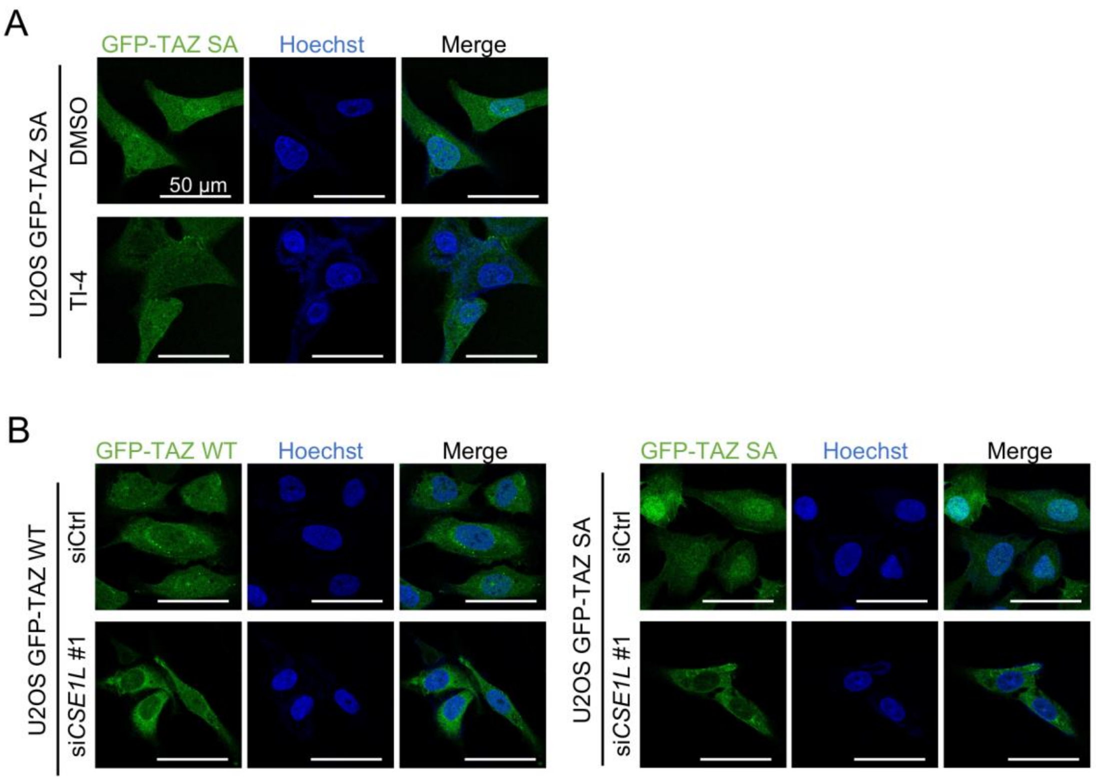

Supplementary Figure 1. The effect of TI-4 and *CSE1L* silencing on the subcellular distribution of TAZ S89A.

(A) U2OS-GFP-TAZ S89A cells were plated 1x10^5 cells /dish in a 35-mm dish. 24 h later, the cells were treated with DMSO or 10 μM TI-4.

(B) U2OS-GFP-TAZ WT and -GFP-TAZ S89A cells were plated 1x10^5 cells/dish in a 35-mm dish, and were transfected control siRNA or *CSE1L* siRNA. 72 h later, the cells were fixed and the nuclei were visualized with Hoechst33342.

The experiments were repeated three times.

Protein	OS	GN	Score	Database	Seq. Coverage(%)	MW(kDa)/pI
CSE1L	Homo sapiens	*CSE1L*	998.4	Swiss-Prot	23.0	110.3 / 5.5

```
        10         20         30         40         50         60         70         80         90        100
MELSDANLQT LTEYLKKTLD PDPAIRRPAE KFLESVEGNQ NYPLLLLTLL EKSQDNVIKV CASVTFKNYI KRNWRIVEDE PNKICEADRV AIKANIVHLM
LSSPEQIQKQ LSDAISIIGR EDFPQKWPDL LTEMVNRFQS GDFHVINGVL RTAHSLFKRY RHEFKSNELW TEIKLVLDAF ALPLTNLFKA TIELCSTHAN
DASALRILFS SLILISKLFY SLNFQDLPEF FEDNMETWMN NFHTLLTLDN KLLQTDDEEE AGLLELLKSQ ICDNAALYAQ KYDEEFQRYL PRFVTAIWNL
LVTTGQEVKY DLLVSNAIQF LASVCERPHY KNLFEDQNTL TSICEKVIVP NMEFRAADEE AFEDNSEEYI RRDLEGSDID TRRRAACDLV RGLCKFFEGP
VTGIFSGYVN SMLQEYAKNP SVNWKHKDAA IYLVTSLASK AQTQKHGITQ ANELVNLTEF FVNHILPDLK SANVNEFPVL KADGIKYIMI FRNQVPKEHL
LVSIPLLINH LQAESIVVHT YAAHALERLF TMRGPNNATL FTAAEIAPFV EILLTNLFKA LTLPGSSENE YIMKAIMRSF SLLQEAIIPY IPTLITQLTQ
KLLAVSKNPS KPHFNHYMFE AICLSIRITC KANPAAVVNF EEALFLVFTE ILQNDVQEFI PYVFQVMSLL LETHKNDIPS SYMALFPHLL QPVLWERTGN
IPALVRLLQA FLERGSNTIA SAAADKIPGL LGVFQKLIAS KANDHQGFYL LNSIIEHMPP ESVDQYRKQI FILLFQRLQN SKTTKFIKSF LVFINLYCIK
YGALALQEIF DGIQPKMFGM VLEKIIIPEI QKVSGNVEKK ICAVGITKLL TECPPMMDTE YTKLWTPLLQ SLIGLFELPE DDTIPDEEHF IDIEDTPGYQ
TAFSQLAFAG KKEHDPVGQM VNNPKIHLAQ SLHKLSTACP GRVPSMVSTS LNAEALQYLQ GYLQAASVTL L
```

Range	Sequence	m/z meas.	z	Δ m/z [ppm]	Modification	Score
18-26	K.TLDPDPAIR.R	499.7711	2	1005.11		44
76-89	R.IVEDEPNKICEADR.V	844.4001	2	-1.57	Carbamidomethyl: 10	32.7
110-120	K.QLSDAISIIGR.E	586.8366	2	2.18		29.4
110-126	K.QLSDAISIIGREDFPQK.W	639.6732	3	-1.34		24
152-159	R.TAHSLFKR.Y	320.5188	3	-0.29		35.2
252-268	K.LLQTDDEEEAGLLELLK.S	643.6721	3	-2.35		37.8
269-288	K.SQICDNAALYAQKYDEEFQR.Y	817.0411	3	-0.05	Carbamidomethyl: 4	53
332-346	K.NLFEDQNTLTSICEK.V	604.6197	3	-1.91	Carbamidomethyl: 13	30.5
347-355	K.VIVPNMEFR.A	552.7957	2	-2.63		32.1
347-372	K.VIVPNMEFRAADEEAFEDNSEEYIRR.D	787.1181	4	-0.81	Oxidation: 6	37.3
356-372	R.AADEEAFEDNSEEYIRR.D	1022.4452	2	-2.88		29.9
372-382	R.RDLEGSDIDTR.R	426.2076	3	-3.55		27.7
373-383	R.DLEGSDIDTRR.R	638.8089	2	-1.77		29
385-391	R.AACDLVR.G	402.7064	2	2.82	Carbamidomethyl: 3	56.2
482-492	K.ADGIKYIMIFR.N	443.2469	3	754.32		32.5
482-492	K.ADGIKYIMIFR.N	448.2434	3	-2.32	Oxidation: 8	23.2
560-574	K.ALTLPGSSENEYIMK.A	826.9131	2	-0.65		24.4
602-607	K.LLAVSK.N	315.7142	2	4.17		21.5
769-777	K.QIFILLFQR.L	589.3571	2	-1.92		26.6
801-816	K.YGALALQEIFDGIQPK.M	881.9651	2	-8.21		40.5
840-848	K.KICAVGITK.L	495.2935	2	-1.51	Carbamidomethyl: 3	21.7
913-925	K.EHDPVGQMVNNPK.I	732.848	2	-0.86		22.1
913-925	K.EHDPVGQMVNNPK.I	740.8442	2	-2.55	Oxidation: 8	32.8

Supplementary Figure 2. The sequence of CSE1L.

Single letters represent amino acid residues. Red letters indicate the sequences identified in Mass spectrometry analysis. The m/z measurement, the modification, and the identification score of each peptide was shown. The mass spectrometry proteomics data have been deposited to the ProteomeXchange Consortium *via* the PRIDE partner repository with the dataset identifier PXD024194 and 10.6019/PXD024194.

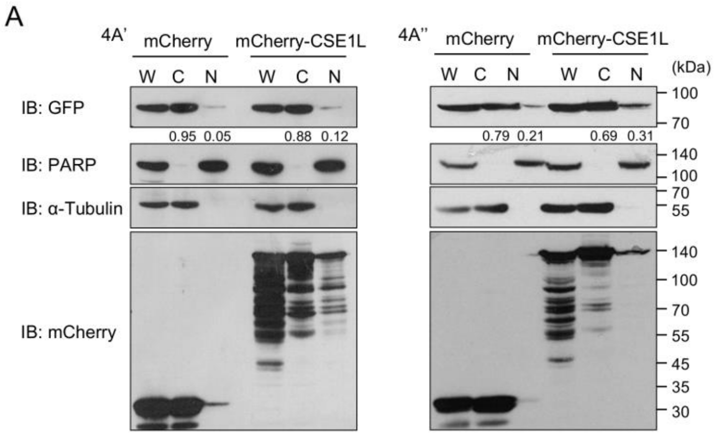

		4A	4A'	4A''	Ave.	SD
mCherry	Cyto.	0.86	0.95	0.79	0.87	0.08
	Nuc.	0.14	0.05	0.21	0.13	0.08
mCherry-CSE1L	Cyto.	0.74	0.88	0.69	0.77	0.1
	Nuc.	0.26	0.12	0.31	0.23	0.1

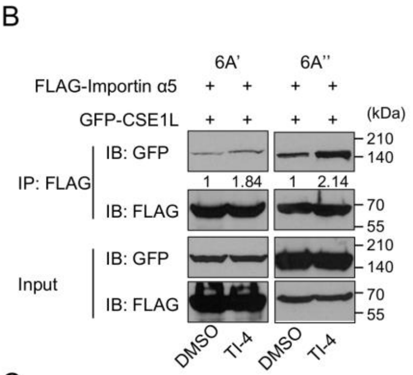

	6A	6A'	6A''	Ave.	SD
DMSO	1	1	1	1	0
TM-4	4.81	1.84	2.14	2.93	1.64

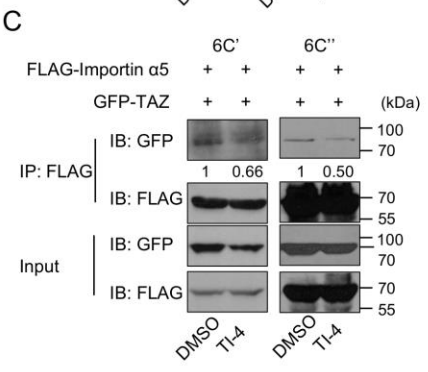

	6C	6C'	6C''	Ave.	SD
DMSO	1	1	1	1	0
TM-4	0.53	0.66	0.50	0.56	0.09

Supplementary Figure 3. Additional results of experiments for Fig. 4C, Fig. 6A, and Fig. 6C.

Two additional results of the experiments shown in Fig. 4C, Fig. 6A, and Fig. 6C are demonstrated to support that the similar results were obtained. The signal intensities are summarized in tables.

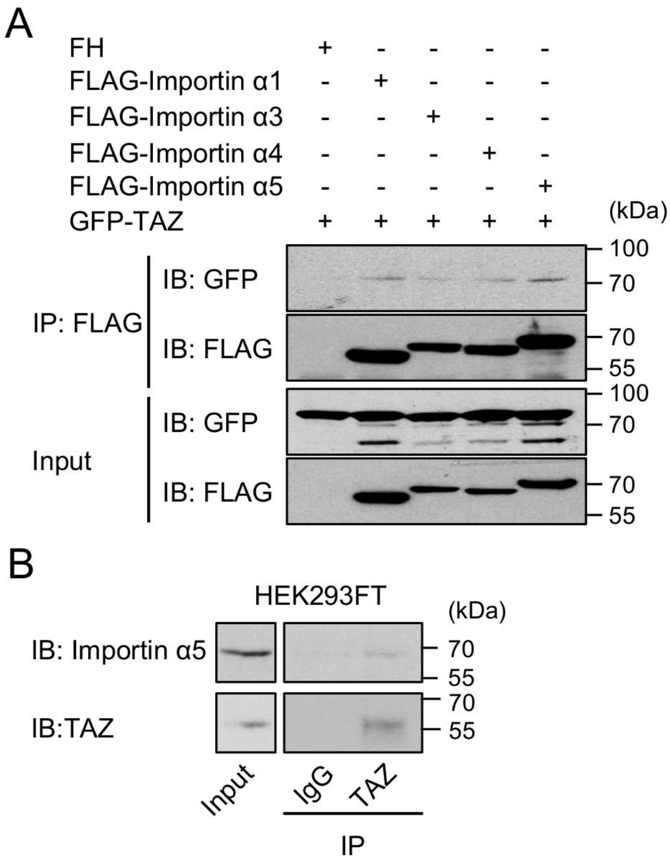

Supplementary Figure 4. Interaction between TAZ and importin α.

(A) HEK293FT cells were transfected with pCIneoGFP-TAZ, pcDNA FLAG-importin α1, α3, α4, and pCIneoFH-importin α5. The immunoprecipitation was performed with anti-DYKDDDDK beads.

(B) Endogenous TAZ was immunoprecipitated with anti-TAZ antibody from HEK293FT cells as described in Experimental procedures. Inputs and immunoprecipitates were immunoblotted with the indicated antibodies. The experiments were repeated three times for (A) and twice for (B).

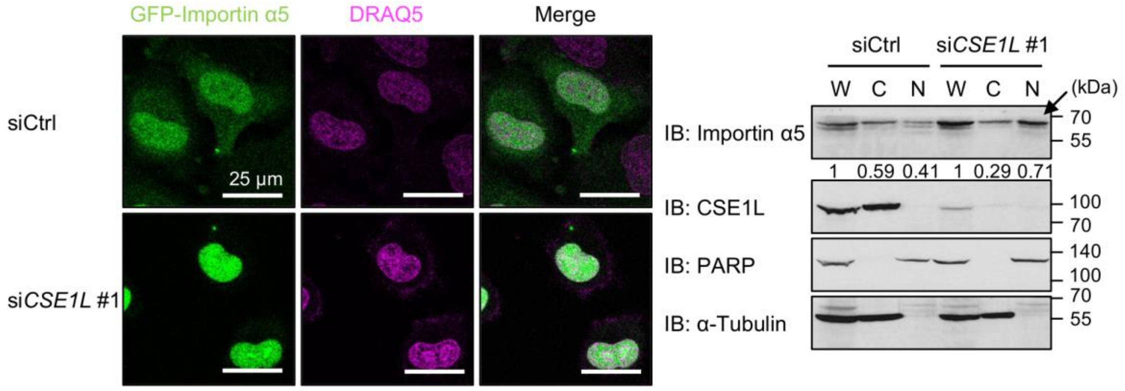

Supplementary Figure 5. *CSE1L* silencing induces the nuclear accumulation of GFP-importin α5.

U2OS cells expressing GFP-importin α5 were transfected with control siRNA or *CSE1L*-targeted siRNA. 48 h later, the cells were observed. 30 min before observation, the nucleus was visualized with DRAQ5™. The subcellular fractionation was performed as described in Experimental procedures. The amount of importin α5 in the nuclear fraction was increased by *CSE1L* silencing (an arrow). The experiments were repeated three times.

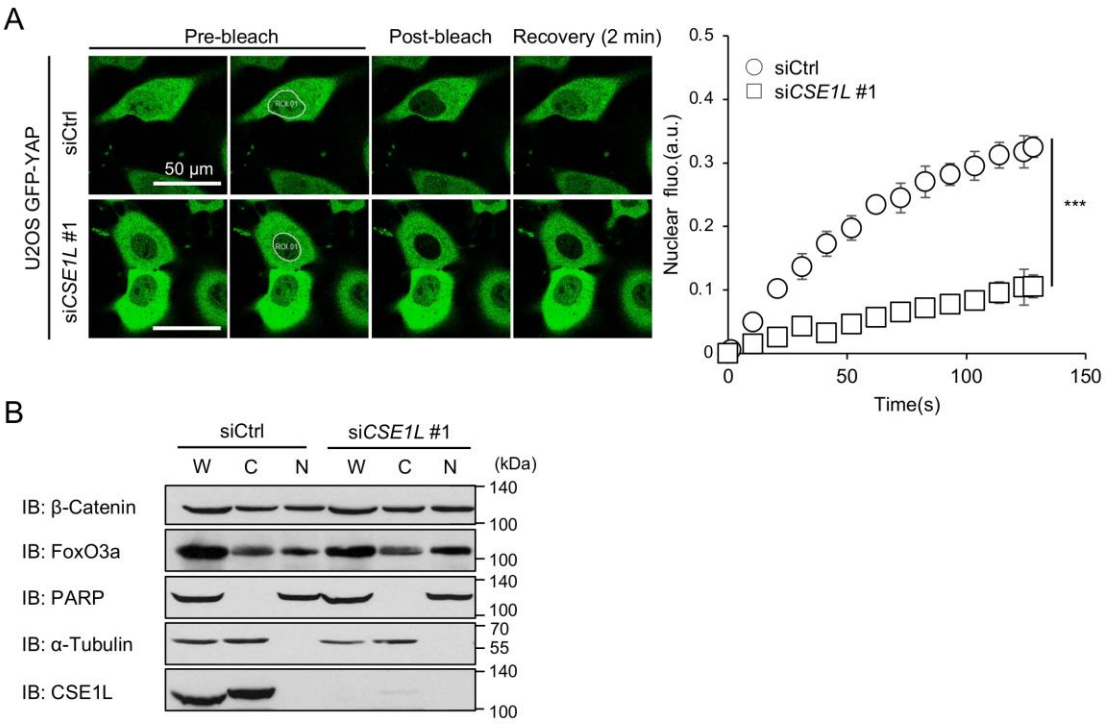

Supplementary Figure 6. CSE1L affects the subcellular distribution of YAP1.

(A) FRAP assay was performed by using U2OSGFP-YAP1 cells as described for U2OS-GFP-TAZ in Figure 5. ***p<0.001

(B) *CSE1L* was knocked down in U2OS cells as described in Figure 4B and the subcellular fractionation was performed. The samples were immunoblotted with the indicated antibodies.

The experiments were repeated three times.

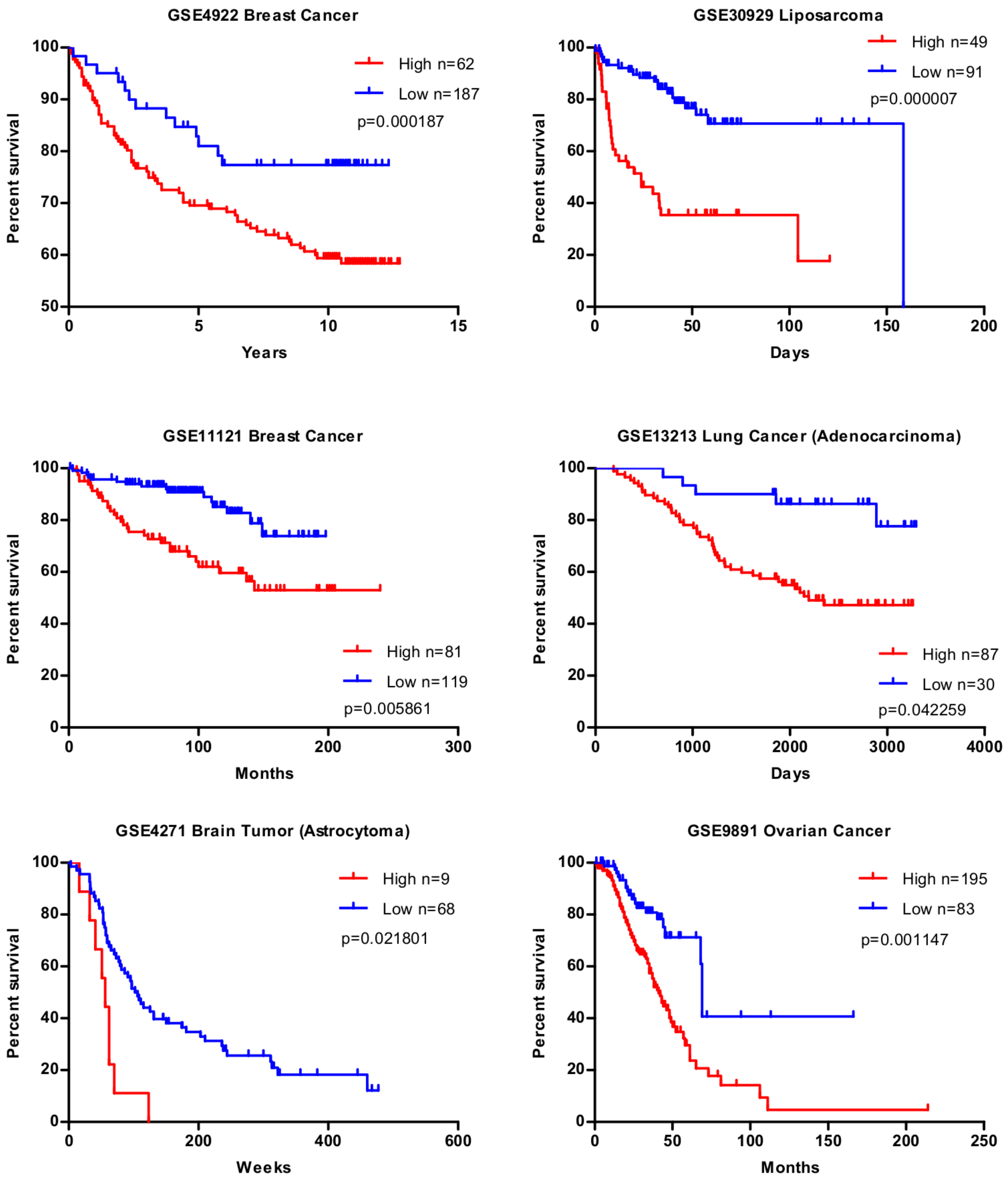

Supplementary Figure 7. High expression of *CSE1L* is associated with poor prognosis in human cancers.

CSE1L expression inversely correlates with prognosis in different types of human cancers. Kaplan-Meier plots were obtained from PrognoScan (http://dna00.bio.kyutech.ac.jp/PrognoScan/).

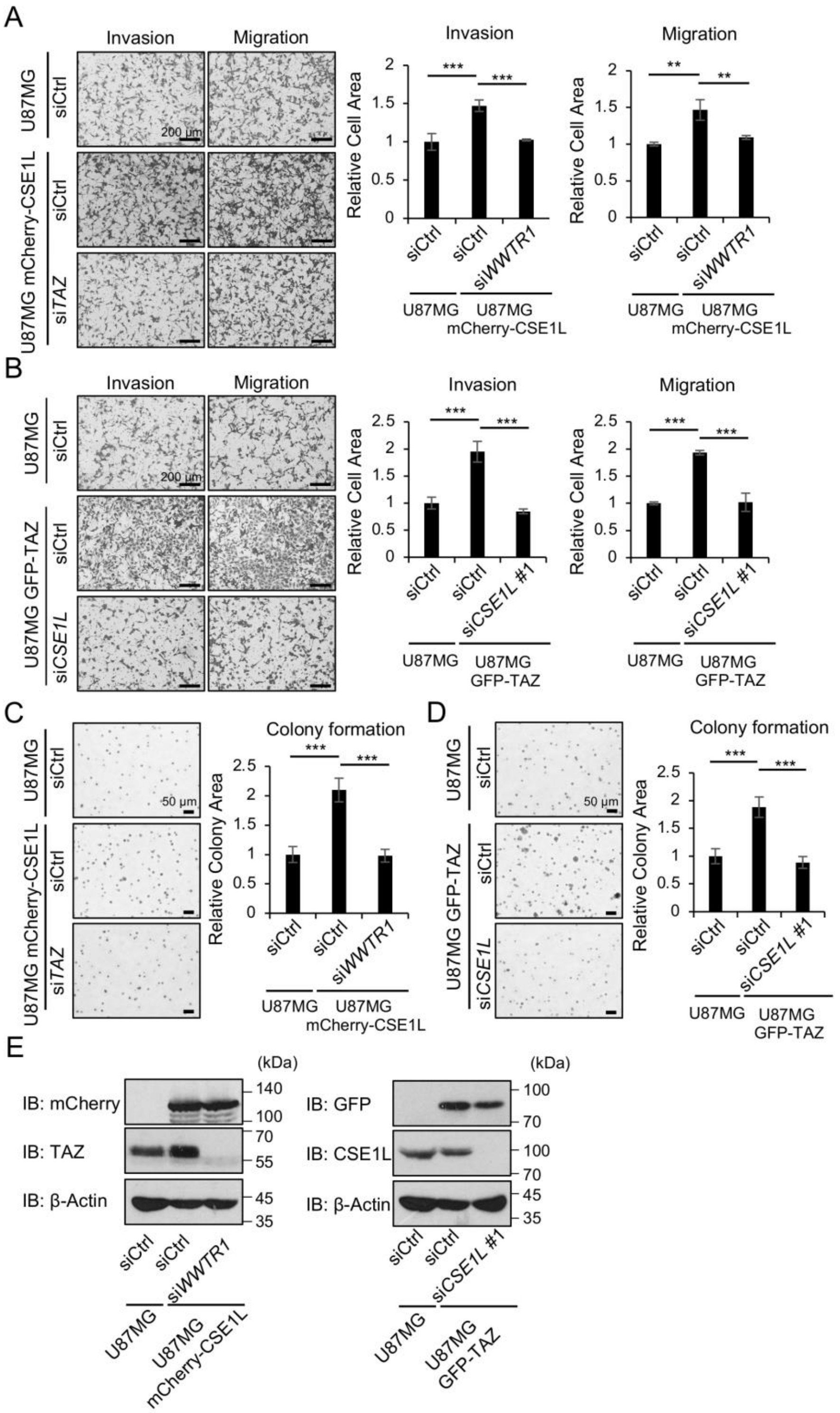

Supplementary Figure 8. CSE1L and TAZ co-operatively promote cell invasiveness, motility, and colony formation in U87MG cells.

The same experiments as described for A549 cells in Figure 7 were performed by using U87MG cells. The experiments were repeated three times.

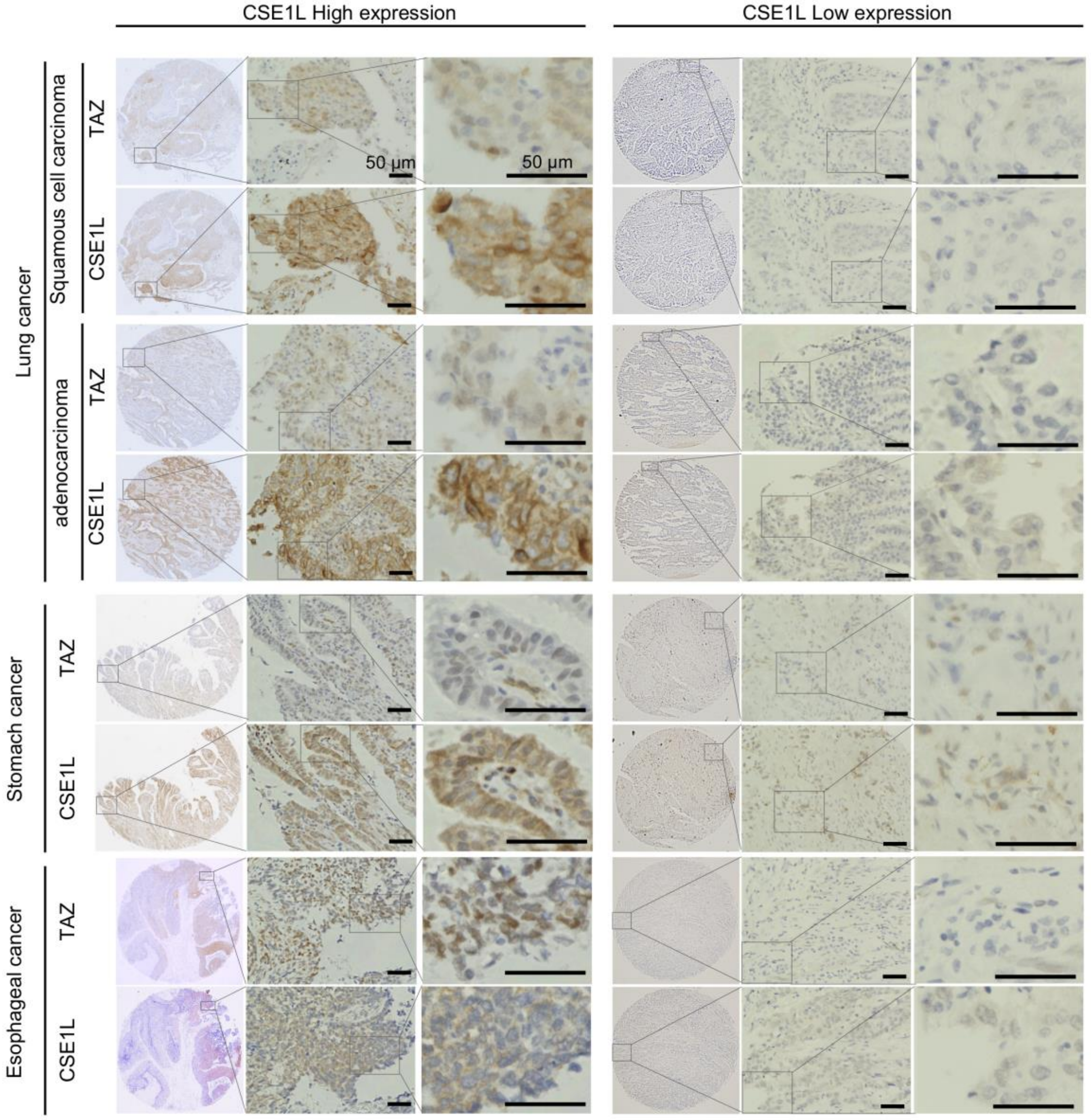

Supplementary Figure 9. Immunohistochemistry of human lung, gastric, and esophageal cancers.

The samples from human cancer microarray were immunostained with anti-TAZ and anti-CSE1L antibodies. The representative images of cancers with high (left) and low (right) expression of CSE1L. Scale bars, 50μm.

Supplementary Table 1 Mass spectrometry search parameters.

Name of peaklist-generating software and release version	Data Analysis 4.3
Name of the search engine and release version	MASCOT 2.7
Name of sequence database searched	Swiss Prot
Release version/date of sequence database searched	SwissProt_2020_06
Number of entries in the database actually searched	563,972
Specificity of all proteases used to generate peptides	Trypsin
Number of missed and/or non-specific cleavages permitted	2
List of all fixed modifications (including residue specificity) considered	Carbamidomethl (C)
List of all variable modifications (including residue specificity) considered	Oxidation (M)
Mass tolerance for precursor ions	30ppm
Mass tolerance for fragment ions	0.07Da
Threshold score/Expectation value for accepting individual spectra	10.0
Peptide identification scores	20.0
